# A dynamic analysis of S&P 500, FTSE 100 and EURO STOXX 50 indices under different exchange rates

**DOI:** 10.1371/journal.pone.0194067

**Published:** 2018-03-12

**Authors:** Yanhua Chen, Rosario N. Mantegna, Athanasios A. Pantelous, Konstantin M. Zuev

**Affiliations:** 1 Institute for Risk and Uncertainty, University of Liverpool, Chadwick Building, L69 7ZF, Liverpool, United Kingdom; 2 Dipartimento di Fisica e Chimica, Università degli Studi di Palermo, Viale delle Scienze Ed. 18, I-90128, Palermo, Italy; 3 Center for Network Science, Central European University, Nador 9, H-1051, Budapest, Hungary; 4 Department of Computer Science, University College London, Gower Street, WC1E 6BT, London, United Kingdom; 5 Department of Mathematical Sciences, University of Liverpool, Peach Street L69 7ZL, Liverpool, United Kingdom; 6 Department of Econometrics and Business Statistics, Monash University, Wellington Rd, Clayton, Victoria, 3800, Australia; 7 School of Management, Shanghai University, No.99 Shangda Road, Shanghai, 200444, China; 8 Department of Computing and Mathematical Sciences, California Institute of Technology, 1200 E. California Blvd. Pasadena, CA, 91125, United States of America; Utah State University, UNITED STATES

## Abstract

In this study, we assess the dynamic evolution of short-term correlation, long-term cointegration and Error Correction Model (hereafter referred to as ECM)-based long-term Granger causality between each pair of US, UK, and Eurozone stock markets from 1980 to 2015 using the rolling-window technique. A comparative analysis of pairwise dynamic integration and causality of stock markets, measured in common and domestic currency terms, is conducted to evaluate comprehensively how exchange rate fluctuations affect the time-varying integration among the S&P 500, FTSE 100 and EURO STOXX 50 indices. The results obtained show that the dynamic correlation, cointegration and ECM-based long-run Granger causality vary significantly over the whole sample period. The degree of dynamic correlation and cointegration between pairs of stock markets rises in periods of high volatility and uncertainty, especially under the influence of economic, financial and political shocks. Meanwhile, we observe the weaker and decreasing correlation and cointegration among the three developed stock markets during the recovery periods. Interestingly, the most persistent and significant cointegration among the three developed stock markets exists during the 2007–09 global financial crisis. Finally, the exchange rate fluctuations, also influence the dynamic integration and causality between all pairs of stock indices, with that influence increasing under the local currency terms. Our results suggest that the potential for diversifying risk by investing in the US, UK and Eurozone stock markets is limited during the periods of economic, financial and political shocks.

## Introduction

The integration among financial markets worldwide has increased markedly of late, due to the rapid flow of capital in the form of direct and indirect investments, and to the globalization of the financial system. In this new era, many countries appear to be more vulnerable than ever before to (global) shocks, as the magnitude and effects of local and international economic, financial and political shocks can be transferred more rapidly in the financial system [1–3]. Furthermore, not only the frequency but also the severity of crises in the markets has increased significantly. In particular, the 2007–09 global financial crisis considerably influenced the international stock markets, and the subsequent European sovereign debt crisis in early 2010 not only had the significant adverse effect on the European stock markets, but also affected those outside of Europe [4, 5]. As a consequence, *integration* and *causality* among those markets have attracted the attention of academia, policy makers and individual investors, as they unveil the complex structure of the global market and, practically, they can influence monetary and fiscal policy coordination and international portfolio diversification [6].

Early research focused mainly on the assets’ price correlation based on stationary returns [7, 8], and correlation has been widely applied to study the mutual interdependence of financial asset returns [9–16]. Song et al. [13] studied the dynamic correlations between 57 international stock market indices, and their results reported both fast and slow dynamics. They argued that the fast dynamics of correlations were associated with the internal or external critical events, and economic and financial shocks, while the slow dynamics reflected consolidation and globalization. Buccheri et al. [14] investigated the correlations between all pairs of stocks traded in the US stock market. They also confirmed that the fast correlations between individual stocks were associated with exogenous or endogenous events, and the slow dynamics indicated that a different degree of diversification of investment was possible. However, the linear correlation is an indicator of co-movement of two time series based on synchronous changes. It might therefore miss long-run relationships occurring on a long time scale [17–19].

The recognition of the non-stationarity of asset prices led to the exploration of possible long-run relations among international stock markets using the cointegration framework to avoid spurious relationship between financial asset series [20–25]. Cointegration is a statistical concept, pioneered by Granger and Engle [20–22]. Generally, two variables are said to be cointegrated when a linear combination of the two is stationary, even though each variable may not be stationary [26]. Empirical studies of the cointegration relationships between some major global stock markets have not provided us with consistent results, since using different data samples, time periods, and data frequencies. For instance, Kanas [27] examined the cointegration relationship between the US and six major European stock markets before and after the 1987 “Black Monday” crash. His results showed no evidence of cointegration among the seven markets. On the other hand, Kasa [28] tested the degree of integration of the US, Japanese, UK, German and Canadian stock markets from 1974 to 1990, and found a single cointegrating vector among the five markets. When Arshanapalli and Doukas [19] studied the dynamic interactions among the US, German, French, UK, and Japanese stock markets, they divided the data sample into two periods, pre- and post-October 1987, to better capture the dynamics of cointegration. Their results showed that, in the later period, the degree of cointegration was significantly greater than in the earlier period. We can also emphasize here that, in this paper, we focus on the dynamic cointegration among the stock market indices, as static cointegration cannot capture the changes in interdependence [2, 29–31]. Moreover, in most of the time-varying cointegration studies, the Johansen test [23–25] has been applied to examine whether one or more cointegrating vectors exist (generally speaking, for more than three variables), while they have not focused on the pairwise dynamic relationship, which is the main contribution of this paper.

The primary feature of cointegrated variables is that their time paths are affected by the extent of any discrepancies from long-run equilibrium. After all, if the system is to return to the long-run equilibrium, the movements of at least some of the variables must respond to the magnitude of the disequilibrium [22, 32]. The Error Correction Model captures this process of adjustment towards an economic equilibrium, and according to Granger’s representation theorem [22, 33], there must be causation in at least one direction among the cointegrated variables in the ECM models. Specifically, the long-term Granger causality are evaluated via the significance of the error correction coefficients in the ECM [34, 35]. The sign and magnitude of the error correction coefficients indicate respectively, that the direction and speed of adjustment towards the long-run equilibrium path. For example, Wahab and Lashgari [36] employed the cointegration technique and ECM to show how the magnitude of adjustments towards the long-run equilibrium in both index and future prices for the S&P 500 and FTSE 100 is formulated for the period of 1988–1992. Their results indicate that future prices exhibit stronger subsequent responses to disequilibrium in the spot prices. In Arshanapalli and Doukas [19], despite that the cointegration relationships existed between the pairwise stock exchange markets of US and France, US and Germany, US and UK in the post-October 1987 period, the insignificant adjustment coefficients of the error correction terms implies that the equilibrium error cannot be used to predict next period’s stock market price changes. Olawale and Taofik [37] showed statistical significant long-run relationship between macroeconomic variables and the FTSE 100 and S&P 500 stock market indices, their results further indicated that US stock market has a quicker speed of adjustment to its long-run equilibrium than that of UK stock market.

Furthermore, Alexander [38, 39] and Miao [40] argued that cointegration and correlation are somewhat related concepts but that some differences exist. For instance, they found that high correlation of asset returns does not necessarily indicate high cointegration in asset prices, and vice versa. Actually, correlation is a short-run measure of co-movement, and is liable to instability over time. On the other hand, cointegration measures the long-run co-movements in asset prices, which may occur even during periods when correlation appears to be low. In this paper, the differences and similarities between the correlation, the cointegration and ECM-based long-run Granger causality of international stock markets are studied using a dynamic framework that considers the various economic, financial and political shocks in the economy.

Since the replacement of fixed exchange rates with floating ones in the 1970s, economic and financial crises in the markets have led currencies to fluctuate substantially. In particular, Eun and Shim [18] examined the world’s nine developed stock markets’ interactions in terms of local currency units to avoid the effect of currency devaluation and appreciation after the occurrence of crises. Alexander and Thillainathan [41] found evidence of cointegration when the stock market indices were expressed in local currency terms. Additionally, Voronkova [42] showed a higher degree of cointegration among stock markets in central Europe, France, Germany, UK and US under the local currencies. Furthermore, the effects of currency devaluation or appreciation after the occurrence of crises (or unexpected events) was no longer present when the stock indices they used in their analyses had been converted to the same currency [43–45]. Hyde et al. [46] found evidence of asymmetries in conditional volatility for local currency returns, while the asymmetry disappeared among the Asian, US and European stock markets when measured using the US dollar currency (It should be mentioned here that Gilmore et al. [30] commented that, when all indices are expressed in US dollar terms, the results of the study are particularly useful to the US, but also to international investors.). On the contrary, Roll [47] argued that such a transformation did not entirely eliminate the influence of exchange rates (see also [48] and [49]). Thus, changes in exchange rates might affect the short-term co-movement behavior between two international stock markets but it has not yet been fully investigated how the dynamic framework might influence them. Hence, in the present paper, we intend to fill this gap and answer the following four fundamental questions:

- How does the pairwise dynamic long-run cointegration changed between international stock indices?
- How does the long-run ECM-based Granger causality varied over time between cointegrated stock indices?
- What are the differences and similarities between the dynamic correlation, cointegration and long-run ECM-based Granger causality?
- How do the different exchange rates affect both dynamic correlation, cointegration and long-run ECM-based Granger causality?

With these concerns in mind, the objective of this work is to study the impact of economic, financial, and political episodes on the S&P 500, FTSE 100 and EURO STOXX 50 stock market indices, using the correlation, cointegration and ECM-based long-run Granger causality tests in a dynamic framework. Additionally, we study whether changes in the foreign exchange rates affect the pairwise integration and causality behavior of the stock markets. Overall, the main contributions of this study are as follows. Firstly, we employ a rolling-window technique by choosing a window size of one year for the correlation and cointegration tests for the S&P 500, FTSE 100 and EURO STOXX 50 (EURO STOXX 50 was launched on February 26th, 1998) indices from January 1st, 1980 to December 29th, 2015. In particular, the rolling-window analysis gives us the opportunity to compare the levels of correlation and cointegration relations before and after specific episodes of financial distress over that period. Second, the rolling-window dynamic ECM-based long-run Granger causality tests provide more interesting results not only for the interaction detection, but also for the directed causal relations over time. Third, during the periods of economic, financial and political shocks, the difference and similarity of dynamic correlation, cointegration and ECM-based long-run Granger causality between the pairs of stock market indices are detected. Finally, unlike previous studies in the corresponding literature, in this study, the dynamic correlation, cointegration and ECM-based long-run Granger causality are measured using common and domestic currency terms. Thus, we are able to investigate how the fluctuation of exchange rates influences the *integration* and *causality* behavior between all the combinations of pairs from those three stock market indices from 1980 to 2015.

## Materials and methods

### Data

We choose three international stock market indices in this study, to cover the three major, most liquid and developed financial markets in the world, i.e., US, UK and Eurozone. The data consist of two groups: three stock indices, the S&P 500, FTSE 100 and EURO STOXX 50, and three exchange rates, the USD (US dollar), GBP (UK pound) and EUR (Euro). All data are from Thomson Reuters DataStream.

**Fig 1:**
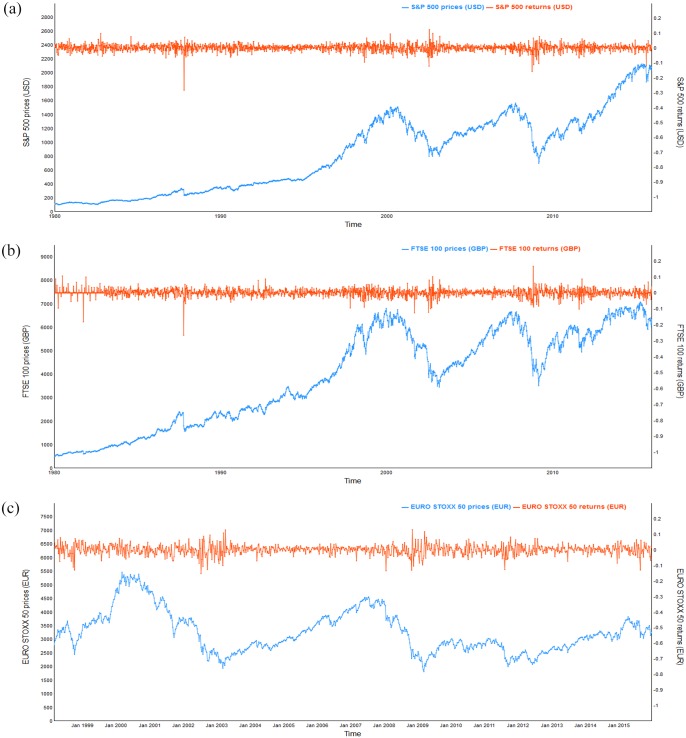
Time variations in weekly stock price indices and returns of S&P 500, FTSE 100 and EURO STOXX 50 based on local currency terms. (a) Weekly stock price indices and returns of S&P 500 from 1980–2015. (b) Weekly stock price indices and returns of FTSE 100 prices and returns from 1980–2015. (c) Weekly stock price indices and returns of EURO STOXX 50 prices and returns from 1998–2015.

In order to avoid the “non-synchronous trading effect” [18, 50], which is related to the fact that not all the markets are open during the same hours of the day, we choose to use weekly data. The data range from January 1st, 1980 to December 29th, 2015, apart from that for the EURO STOXX 50 index, for which data was available from February 26th, 1998. The samples of the S&P 500 and FTSE 100 consist of 1879 observations each, and that of the EURO STOXX 50 index contains 932 observations. Fig 1 plots the original stock price index and returns for the S&P 500, FTSE 100 and EURO STOXX 50, respectively. Over the past 35 years from 1980 to 2015, the price indices of the S&P 500 and FTSE 100 appear to have stochastic trends and seem to reveal similar behavior from the beginning until 2009. Two peaks occurred, in 2000 and 2007, followed by sharp declines in 2001 and 2008 for all three indices. Then, the S&P 500 recovered strongly from 2009 until the end of December 29th, 2015, while the performance of the FTSE 100 and EURO STOXX 50 indices lagged behind that of the S&P 500 but exhibited similar increasing trends. Furthermore, from the movement of the returns in Fig 1, we can deduce that the downward movements of the S&P 500, FTSE 100 and EURO STOXX 50 tend to be associated with large returns. Table 1 provides the name and date of each economic and financial shock that occurred around the world between 1980 and 2015. In addition, to study how the exchange rates fluctuations affect the pairwise interdependence of stock markets, the pairs of stock price indices, namely, S&P 500 with FTSE 100, S&P 500 with EURO STOXX 50, and FTSE 100 with EURO STOXX 50, each of those pairs are converted using the same currency (i.e., fixing the exchange rates fluctuations) and their domestic currencies (i.e., permitting exchange rates fluctuations). The details of our sample are reported in Table 2.

**Table 1:** List of economic, financial and political shocks during 1980–2015.

Period	Name of the shocks	Date
1	Early 1980s recession in the UK	January 1st, 1980–March 31st, 1981
2	Early 1980s recession in the US	July 1981–November 1982
3	1982 Latin American debt crisis	August 1982
4	Economic recovery of the US and UK	From December 1982
5	1984–85 UK miners’ strike	March 5th, 1984–May 3rd, 1985
6	Beginning of the US saving & loan crisis	March 5th, 1985
7	1985–87 US economic crisis after Palza Accord	December 22nd, 1985–1987
8	1987 Lawson Boom in the UK	March 1987
9	1987 “Black Monday” stock market crash	October 17th, 1987
10	1989 mini-crash of stock market	October 13th, 1989
11	1990 Japanese asset bubble collapse	December 29th, 1989
12	1990 Gulf War	August 2nd, 1990–February 28th, 1991
13	Early-1990s recession in the US & UK	July 1990–March 1991, US (July 1990–September 1991, UK)
14	1991 European Union established	December 31st, 1991
15	1992 “Black Wednesday” in the UK	September 16th, 1992
16	1992–93 European currency crisis	January 1st, 1993
17	1994 Mexico peso crisis	December 20th, 1994
18	1995-96 US government shut-down	November 13th, 1995–January 6th, 1996
19	1997 Asian financial crisis	July 2nd, 1997
21	1998 Russian financial crisis	August 17th, 1998
22	1999 Euro introduced	January 1st, 1999
23	1999 Kosovo War	March 24th, 1999
24	2000 bursting of dot-com bubble	March 10th, 2000
25	2001 Turkish economic crisis	February 19th, 2001
26	Early-2000s recession in the US	March 2001
27	9/11 Attacks	September 11th, 2001
28	2001 US war in Afghanistan	October 7th, 2001
29	2002 stock market downturn	October 9th, 2002
30	2003 US war in Iraq	March 20th, 2003
31	Beginning of US housing bubble of 2004–06	February 2004
32	Collapse of US housing bubble in mid-2006	June 2006
33	Origin of 2007 sub-prime mortgage crisis	April 2nd, 2007
34	US recession of Dec 2007–Jun 2009	December 2007
35	2008 Lehman Brothers collapse	September 16th, 2008
36	US QE1 announced	November 25th, 2008–March 31th, 2010
37	UK QE1 announced	March 5th, 2009–February 4th 2010
38	US QE1 extension	March 18th, 2009
39	2009 Dubai debt standstill	November 27th, 2009
40	2010 European sovereign debt crisis	April 27th, 2010
41	US QE2 announced	November 3th, 2010–June 3th, 2011
42	2011 Stock Market Fall	August 1st, 2011
43	US Operation Twist announced	September 11th, 2011–September 13th 2012
44	UK QE2 announced	October 6th, 2011–May 10th, 2012
45	UK QE3 announced	July 5th, 2012–November 5th, 2012
46	US QE3 announced	September 13th, 2012–October 31th, 2014
47	US QE3 extended & Operation Twist ends	December 12th, 2012
48	US QE3 taper announced	December 18th, 2013
49	2013 US debt-ceiling crisis	January 1st, 2013
50	2014 Russian financial crisis	December 16th, 2014
51	EU QE announced	January 22nd, 2015–present
52	2015–16 Chinese stock market turbulence	June 12th, 2015
53	2015–16 US stock market selloff	August 15th, 2015

**Table 2:** The three pairs of indices out of S&P 500, FTSE 100 and EURO STOXX 50, and the different currency terms used.

Stock Market Indices	Common Currency	Common Currency	Domestic Currencies
S&P 500 vs. FTSE 100	USD/USD	GBP/GBP	USD/GBP (GBP/USD)
S&P 500 vs. EURO STOXX 50	USD/USD	EUR/EUR	USD/EUR (EUR/USD)
FTSE 100 vs. EURO STOXX 50	GBP/GBP	EUR/EUR	GBP/EUR (EUR/GBP)

### Methods

This section describes the steps to measuring the time-varying pairwise correlation, cointegration and ECM-based long-run Granger causality of the stock markets. For the rolling-window technique, first, we choose a rolling window of size *l*, which is the number of observations per rolling window, and we set the number of increments between successive rolling windows. Then, the entire sample *T* is converted into *N* = *T* − *l* + 1 sub-samples. Thus, the first rolling window contains observations for the first period through *l*, the second rolling window contains observations for the second period through *l* + 1, and so on [51].

#### Rescaling the original stock index series

Since our stock market indices have different scales, they must be rescaled so as to be comparable. Thus, the first step is to calculate the percentage changes of each stock index series, which are given by

$$\begin{array}{c} \Delta_{i}\left(t\right)=\frac{P_{i}\left(t\right)}{P_{i}\left(t-1\right)},\;\text{for}\;\text{all}\;t\geq 2, \end{array}$$ (1)

where *P*_*i*_(*t*) is the price of index *i* in week *t*. For the rescaled index series *R*_*i*_(*t*), we set the first entry in each series to be *R*_*i*_(1) = 1, and then *R*_*i*_(*t*) is expressed, for all subsequent entries in each series, by

$$\begin{array}{c} R_{i}\left(t\right)=R_{i}\left(t-1\right)*\Delta_{i}\left(t\right),\;\text{for}\;\text{all}\;t\geq 2. \end{array}$$ (2)

After rescaling the original stock index series, we eventually transform them into their returns and natural logarithms for the correlation and cointegration test, respectively.

#### Rolling-window correlation test

To detect the interdependencies between variables, we apply the conventional Pearson correlation coefficient [52]. The analysis is based on the weekly logarithmic return after rescaling, which is given by Eq (3) for each stock index *i*:

$$\begin{array}{c} r_{i}\left(t\right)=\ln R_{i}\left(t\right)-\ln R_{i}\left(t-1\right), \end{array}$$ (3)

where *R*_*i*_(*t*) is the price of index *i* in week *t* after rescaled by Eqs (1) and (2). Then, in each time window, the Pearson correlation coefficient between returns *i* and *j* is given by

$$\begin{array}{c} C_{i,j}=\frac{\langle \left[r_{i}\left(t\right)-\mu_{i}\right]\left[r_{j}\left(t\right)-\mu_{j}\right]\rangle }{\sigma_{i}\sigma_{j}}, \end{array}$$ (4)

where *μ*_*i*_ and *μ*_*j*_ are the mean of the two returns *i* and *j*, *σ*_*i*_ and *σ*_*j*_ are the standard deviation of the *i* and *j*, respectively.

#### Rolling-window cointegration test

The cointegrated variables must obey an equilibrium relationship in the long run, although they may diverge substantially from that equilibrium in the short run. Based on the traditional Engle-Granger [22, 53] cointegration test, our methodology consists of the following two steps to examine cointegration for the non-stationary financial asset price series:

**Step 1:** Rolling-window Unit Root Tests

Before we proceed further, we first perform unit root tests for each stock market index to identify whether they are *I*(1) (the integration of order one is denoted by *I*(1) and a stationary process is denoted by *I*(0)) [20–22]. The stationarity is tested after taking the first difference by implementing the most popular Dickey-Fuller (hereafter referred to as DF) [54], augmented Dickey-Fuller (hereafter referred to as ADF) and Phillips-Perron (hereafter referred to as PP) unit root tests [55].

The DF and ADF tests are based on the following regression:

$$\begin{array}{c} \Delta y_{t}=\beta^{\prime }D_{t}+\gamma y_{t-1}+\sum_{i=1}^{p}\delta_{i}\Delta y_{t-i}+\varepsilon_{t}, \end{array}$$ (5)

where *δ*_*i*_ equals zero for the DF tests, *y*_*t*_ is the logarithm of the rescaled index series for time period *t*, *D*_*t*_ is a vector of deterministic terms (constant, trend etc.), *γ* is the coefficient presenting the process root, $\sum_{i=1}^{p}\delta_{i}\Delta y_{t-i}$ are lagged values of *y*_*t*_, *p* is the lag order of the auto-regressive process, and *ε*_*t*_ is the error term that should be white noise in our case. Here, the lag length *p* is decided by $p_{\max}=[12{\left(\frac{T}{100}\right)}^{\frac{1}{4}}]$, where *T* is the sample size of an index series. Then, we set *p* = *p*_*max*_ and perform the ADF test to minimize the Schwarz information criterion [56] (hereafter referred to as SIC). The null hypothesis is that the stock market indices have a unit root (*H*_0_: *γ* = 0), against the alternative that they do not (*H*_0_: *γ* < 0) by means of the *t*-statistics:

$$\begin{array}{c} t_{\gamma =0}=\frac{\hat{\gamma }}{SE(\hat{\gamma })}, \end{array}$$ (6)

where $SE(\hat{\gamma })$ is the standard error of the OLS estimate $\hat{\gamma }$ in the Eq (5). Different from the DF and ADF tests, the advantage of the PP tests over them is that the PP tests are robust to general forms of heteroskedasticity in the error term *u*_*t*_. Another advantage is that the user does not have to specify a lag length for the test regression.

$$\begin{array}{c} \Delta y_{t}=\beta^{\prime }D_{t}+\pi y_{t-1}+u_{t}, \end{array}$$ (7)

where *u*_*t*_ is *I*(0) and may be heteroskedastic. The PP tests correct for any serial correlation and heteroskedasticity in the errors *u*_*t*_ of the test regression by directly modifying the *t*-statistics *t*_*π* = 0_. The modified *t*-statistic, denoted *Z*_*t*_, is given by

$$\begin{array}{c} Z_{t}={\left(\frac{{\hat{\sigma }}^{2}}{{\hat{\lambda }}^{2}}\right)}^{1/2}\cdot t_{\pi =0}-\frac{1}{2}\left(\frac{{\hat{\lambda }}^{2}-{\hat{\sigma }}^{2}}{{\hat{\lambda }}^{2}}\right)\cdot \left(\frac{T\cdot SE(\hat{\pi })}{{\hat{\sigma }}^{2}}\right) \end{array}$$ (8)

The terms *σ*^2^ and λ^2^ are consistent estimates of the variance parameters, and $\sigma^{2}=\lim_{T\to \infty }T^{-1}\sum_{t=1}^{T}E\left[\varepsilon_{t}^{2}\right]$, $\lambda^{2}=\lim_{T\to \infty }T^{-1}\sum_{t=1}^{T}E\left[T^{-1}S_{T}^{2}\right]$ (where $S_{T}=\sum_{t=1}^{T}\varepsilon_{t}$), respectively. The null hypothesis for the PP tests is that *H*_0_: *π* = 0 (stock market index has a unit root) against the alternative *H*_0_: *π* < 0 (stock market index is stationary).

Once we have established that all stock indices are *I*(1) in each time window, the rolling Engle-Granger cointegration test could be implemented.

**Step 2:** Rolling-window Engle-Granger Two-step Cointegration Tests

We apply the Engle-Granger cointegration test [22], which is a two-step process. First, the determination of the linear relationship is required, and then the stationarity testing on the residuals follows. As the Engle-Granger cointegration procedure is sensitive to the choice of dependent variable [22, 57], we use the forward and reverse cointegrating regressions [22, 36, 58] for the two *I*(1) variables:

$$\begin{array}{c} y_{t}=\alpha_{1}+\beta_{1}x_{t}+u_{1t},\left(\text{when}\;y_{t}\;\text{is}\;\text{the}\;\text{dependent}\;\text{variable}\right), \end{array}$$ (9)

$$\begin{array}{c} x_{t}=\alpha_{2}+\beta_{2}y_{t}+u_{2t},\left(\text{when}\;x_{t}\;\text{is}\;\text{the}\;\text{dependent}\;\text{variable}\right), \end{array}$$ (10)

where *y*_*t*_ and *x*_*t*_ are the logarithms of the rescaled stock index series for time period *t*, *α*_1_ and *α*_2_ are intercept constants, *β*_1_ and *β*_2_ are cointegration coefficients. Particularly, *u*_1*t*_ and *u*_2*t*_ are long-run equilibrium error as the measurement for the deviation of *y*_*t*_ and *x*_*t*_ from the cointegration relationship, respectively. If ${\hat{u}}_{1t}∼I\left(0\right)$ and ${\hat{u}}_{2t}∼I\left(0\right)$, then two *I*(1) variables *y*_*t*_ and *x*_*t*_ are said to be cointegrated. However, in the financial market, even if there is cointegration between *y*_*t*_ and *x*_*t*_ in the long term, there could be disequilibrium caused by disturbances in the short term. Then the short-run deviation from the long-run equilibrium relationship can be captured by the Error Correction Model [20, 22, 33] to guarantee that the two variables do not drift too far apart [59].

Here, the second step of the Engle-Granger cointegration tests, the stationarity test of ${\hat{u}}_{1t}$ and ${\hat{u}}_{2t}$ are employed both the DF and PP tests to make the results more convinced. The null and alternative hypotheses for stationarity of residuals are given by:

$$\begin{array}{c} \begin{array}{c} H_{0}:{\hat{u}}_{1t}\left({\hat{u}}_{2t}\right)\;\text{is}\;\text{non}\;\text{stationary}\Leftrightarrow y_{t}\left(x_{t}\right)\;\text{does}\;\text{not}\;\text{cointegrate}\;\text{with}\;x_{t}\left(y_{t}\right);\\ H_{1}:{\hat{u}}_{1t}\left({\hat{u}}_{2t}\right)\;\text{is}\;\text{stationary}\Leftrightarrow y_{t}\left(x_{t}\right)\;\text{cointegrates}\;\text{with}\;x_{t}\left(y_{t}\right). \end{array} \end{array}$$ (11)

#### Rolling-window Error Correction Model

According to Granger Representation Theorem [22], once two variables are found to be cointegrated, there necessarily exists causality (in the Granger sense, not in the structure sense) at least one direction. The direction of the Granger causality can be further ascertained using the ECM between cointegrated variables (*y*_*t*_, *x*_*t*_):

$$\begin{array}{c} \Delta y_{t}=\alpha_{1}+\sum_{i}^{p}\beta_{1i}\Delta y_{t-i}+\sum_{i}^{q}\gamma_{1i}\Delta x_{t-i}+\delta_{y}ECT_{y,t-1}+\eta_{1t}, \end{array}$$ (12)

$$\begin{array}{c} \Delta x_{t}=\alpha_{2}+\sum_{i}^{p}\beta_{2i}\Delta y_{t-i}+\sum_{i}^{q}\gamma_{2i}\Delta x_{t-i}+\delta_{x}ECT_{x,t-1}+\eta_{2t}, \end{array}$$ (13)

where

$$\begin{array}{c} ECT_{y,t-1}={\hat{u}}_{1,t-1}=y_{t-1}-\left[{\hat{\alpha }}_{1}+{\hat{\beta }}_{1}x_{t-1}\right], \end{array}$$ (14)

$$\begin{array}{c} ECT_{x,t-1}={\hat{u}}_{2,t-1}=x_{t-1}-\left[{\hat{\alpha }}_{2}+{\hat{\beta }}_{2}y_{t-1}\right], \end{array}$$ (15)

The Eqs (12) and (13) imply that changes in the dependent variable are a function of the magnitude of disequilibrium in the cointegrating relationship (capture by the Error Correction Term (hereafter referred to as ECT)) as well as changes in other explanatory variables, respectively. Specifically, the Δ*y*_*t*_ and Δ*x*_*t*_ represent the first differences between these variables and capture their short-run disturbances, the coefficients *α*_1_, *α*_2_, *β*_1*i*_, *β*_2*i*_, *γ*_1*i*_ and *γ*_2*i*_ are the coefficient estimates for independent variables, *η*_1*t*_ and *η*_2*t*_ are disturbance terms. The lagged *ECT*_*y*,*t*−1_ and *ECT*_*x*,*t*−1_ in Eqs (14) and (15) refer to the lagged residual derived from long-run cointegrating relationship. Generally, in long-run equilibrium, the ECTs are equal to zero. However, if *y*_*t*_ and/or *x*_*t*_ deviate from the long-run equilibrium in the short run, the ECTs will not equal to zero and each dependent variable adjusts to partially restore the equilibrium relations caused by the disequilibrium of ECTs. The speed of adjustment parameters *δ*_*y*_ (*δ*_*x*_) of the lagged ECTs indicates how quickly the dependent variable returns to its long-run equilibrium after a temporary departure creased by the *x*_*t*_ (*y*_*t*_) in the Eq 9(Eq 10) when they are statistically significant through the *t*-test [33, 34, 60–62]

In addition to indicating the direction of causality amongst variables, the ECM approach allows for testing both short run and long run Granger-causality [60, 61], which is more robust and more powerful than the standard Granger causality test [33, 63]. According to Granger [34], the long-term causality is evaluated via the significance of the *δ*_*y*_ and *δ*_*x*_ using the standard *t*-statistic, respectively. The null hypothesis of long-run non Granger causality from *x*_*t*_ to *y*_*t*_ is given by *δ*_*y*_ = 0 in Eq (12), on the contrary, the null hypothesis of long-run non causality from *y*_*t*_ to *x*_*t*_ is given by *δ*_*x*_ = 0 in Eq (13).

#### Benjamini and Hochberg false discovery rate control

For each pairwise test of stock market indices, determining whether an observed result is statistically significant, requires comparing the corresponding statistical confidence measure (the *p*-value) to a confidence threshold *α* (i.e., 0.01, 0.05 and 0.1). However, as the number of hypotheses increases, so does the probability of incorrect rejections of false positives. Therefore, a multiple hypothesis test correction needs to be done. The False Discovery Rate (hereafter referred to as FDR) is introduced by Benjamini and Hochberg [64], which describes the proportion of false discoveries among total rejections in multiple comparisons. To control and correct the FDR of a family of hypothesis tests, the Benjamini and Hochberg (BH) procedure is utilized [63, 64].

**Step 1**: Calculate the unadjusted *p*-values for *m* hypotheses tests and sort them in ascending order, *p*(1) ≤ *p*(2) ≤ … ≤ *p*(*m*). Set the smallest *p*-value has a rank of *i* = 1, then next smallest has *i* = 2, etc.
**Step 2**: Compare each individual *p*-value to its BH critical value, $\alpha \times \frac{i}{m}$, where *i* is the rank, *m* is the total number of tests, and *α* is the FDR you choose.
**Step 3**: Define *k* to be the largest rank *i* for which $p\left(i\right)\leq \alpha \times \frac{i}{m}$. Declare all tests of rank 1, 2, …, *i* as significant with *p*-values smaller or equal to *p*(*k*).

## Results and discussion

A rolling window size of *l* = 48 is chosen as the frame in the paper [51]. By adding one observation at the end and removing the first one, we can divide the full sample into *N* = *T* − 48 + 1 time windows. Then, for each of those rolling time windows, the dynamic analysis of the correlation, unit root tests, cointegration, ECM-based long-run Granger causality test are implemented, respectively.

### Dynamic short-run correlation analysis

**Fig 2:**
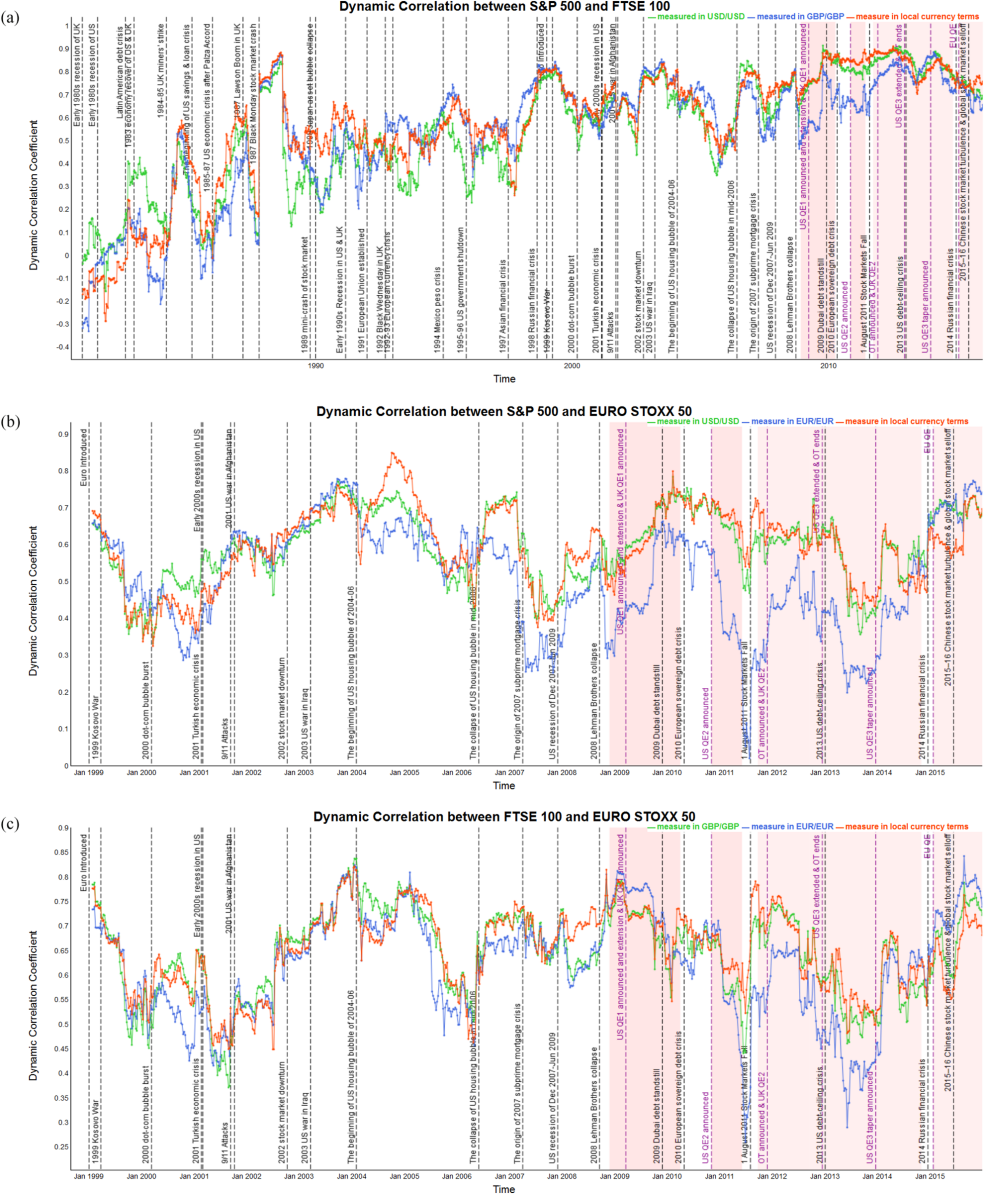
Dynamic correlation between S&P 500, FTSE 100 and EURO STOXX 50 based on common and local currency terms over 1980–2015. The red shading represents implementation of QE policies. (a) Dynamic correlation between S&P 500 and FTSE 100 over 1980–2015. (b) Dynamic correlation between index S&P 500 and EURO STOXX 50 over 1998–2015. (c) Dynamic correlation between FTSE 100 and EURO STOXX 50 over 1998–2015.

In our study, the first measure of the extent of the financial markets’ integration is provided by the correlations estimated using dynamic Pearson correlation analysis. Fig 2(a)–2(c) present the dynamic correlation coefficients for each pair of stock market indices of the S&P 500, FTSE 100 and EURO STOXX 50, when measured in the same and local currency terms from 1980 to 2015. In Table 3, we report the statistical summary in the form of strongest, weakest and average absolute value of correlation coefficients.

**Table 3:** Statistical analysis of dynamic correlation coefficient.

Stock Market Indices	Strongest Coeff	Weakest Coeff	Average Coeff
**S&P 500 vs. FTSE 100**			
Measured in USD/USD	0.917	0.0014	0.544
Measured in GBP/GBP	0.888	0.0003	0.545
Measured in local currencies	0.914	0.0020	0.586
**S&P 500 vs. EURO STOXX 50**			
Measured in USD/USD	0.786	0.3340	0.596
Measured in EUR/EUR	0.781	0.0215	0.515
Measured in local currencies	0.851	0.3260	0.597
**FTSE 100 vs. EURO STOXX 50**			
Measured in GBP/GBP	0.838	0.3730	0.646
Measured in EUR/EUR	0.843	0.2620	0.621
Measured in local currencies	0.822	0.4500	0.652

Observing Fig 2(a)–2(c), the dynamic correlation coefficients between all pairs of stock market indices tend to rise significantly with the economic, financial and political shocks under the influence of high market volatility and uncertainty in the system. However, gradually decreasing during the periods of recovery of the stock market after shocks. Fig 2 also reflects that the dynamic integration between the US and UK stock markets has a consistently positive trend over 1980–2015, compared with the relatively stable and higher-valued trend between the US and Eurozone, UK and Eurozone. Furthermore, in Table 3, we report that the average correlation coefficient between the S&P 500 and FTSE 100 is 0.544 in USD/USD, 0.545 in GBP/GBP, and 0.586 in local currencies. That between the S&P 500 and EURO STOXX 50 is 0.596 in USD/USD, 0.515 in EUR/EUR, and 0.597 in local currency terms, and that between the FTSE 100 and EURO STOXX 50 is 0.646 in GBP/GBP, 0.621 in EUR/EUR, and 0.652 in local currency units. These results suggest that, when measured in local currency terms, the correlation is stronger. In addition, Fig 2 and Table 3 provide evidence that the FTSE 100 and EURO STOXX 50 have the strongest correlation compared with the S&P 500 and FTSE 100 or the S&P 500 and EURO STOXX 50. Besides, the strongest correlation between the S&P 500 and FTSE 100 occurs during period 9, namely, the 1987 “Black Monday” stock market crash. However, the strongest coefficients between the S&P 500 and EURO STOXX 50, and between the FTSE 100 and EURO STOXX 50, both occur during period 31, i.e., at the beginning of 2004–06 US housing asset bubble period. In particular, when we take into account how the changes of exchange rates influence the dynamic correlation coefficients between all three stock market indices, the weakest correlation between the S&P 500 and FTSE 100 is measured in GBP/GBP during periods 4, and 35–49, all of which saw the USD depreciate against the GBP. For the S&P 500 and EURO STOXX 50, we observe the weakest correlation during periods 31–49 when using EUR/EUR, which associated with the USD’s devaluation against the EUR. Furthermore, in periods 42–49, the correlation between the FTSE 100 and EURO STOXX 50 becomes weaker when expressed in EUR/EUR, and again the GBP depreciated against the EUR during that period.

The linear correlation analysis is performed to ascertain the degree of co-movement among the three developed stock markets based on stationary returns. However, such analysis might miss long-run relationships occurring on a long time scale and lack the information of the direction of interaction between international stock markets. For the non-stationary financial asset price series, the implementation of the dynamic cointegration and ECM tests could be used to verify whether a long-term relationship exists, and to examine the long-run Granger causality, respectively.

### Dynamic unit root test analysis

**Fig 3:**
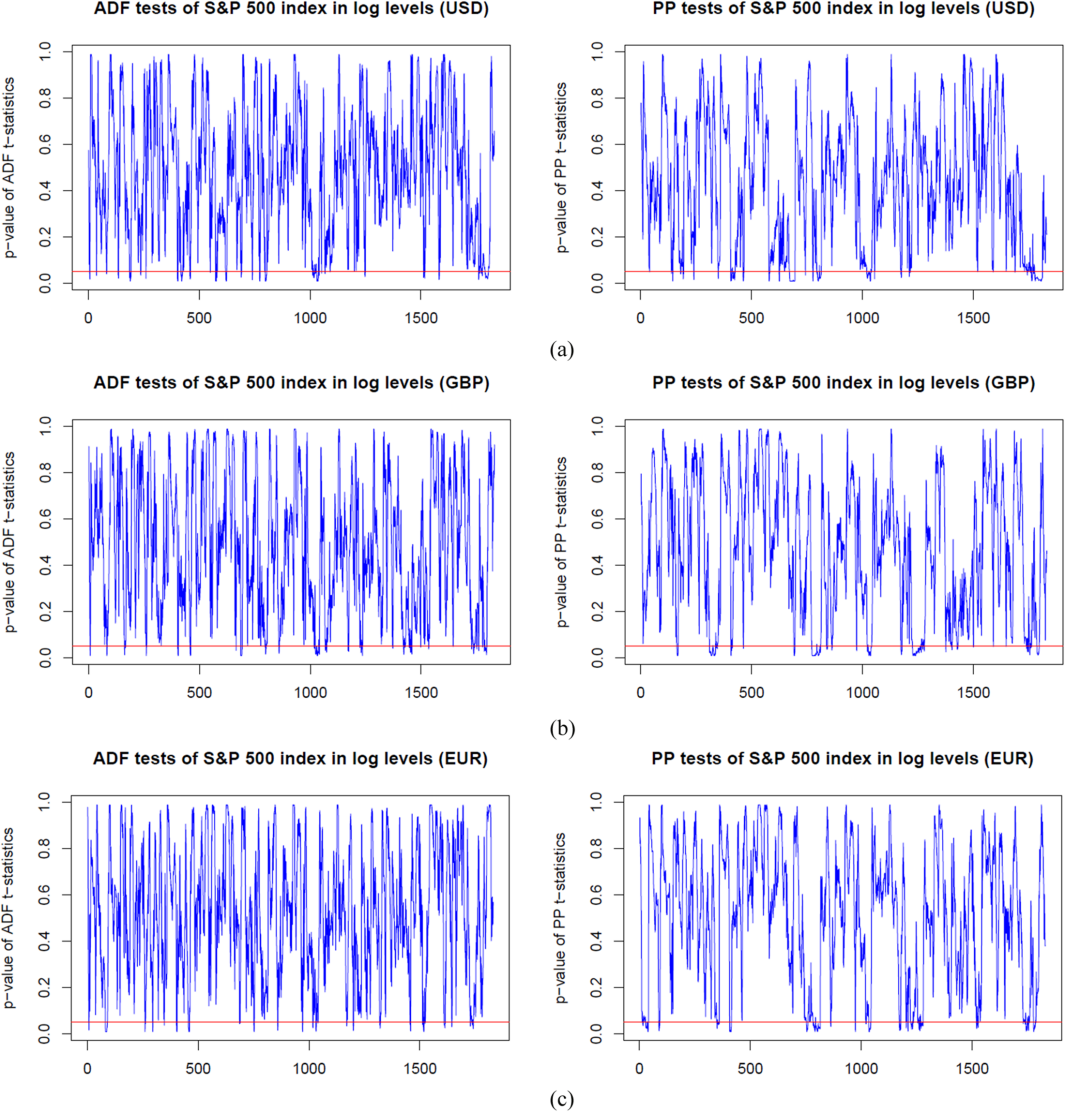
*p*-values from dynamic ADF and PP unit root tests of the S&P 500 index based on USD, GBP and EUR respectively, in log levels. The red line indicates 5% statistical significance level. (a) S&P 500 index in log level (USD). (b) S&P 500 index in log level (GBP). (c) S&P 500 index in log level (EUR).

**Fig 4:**
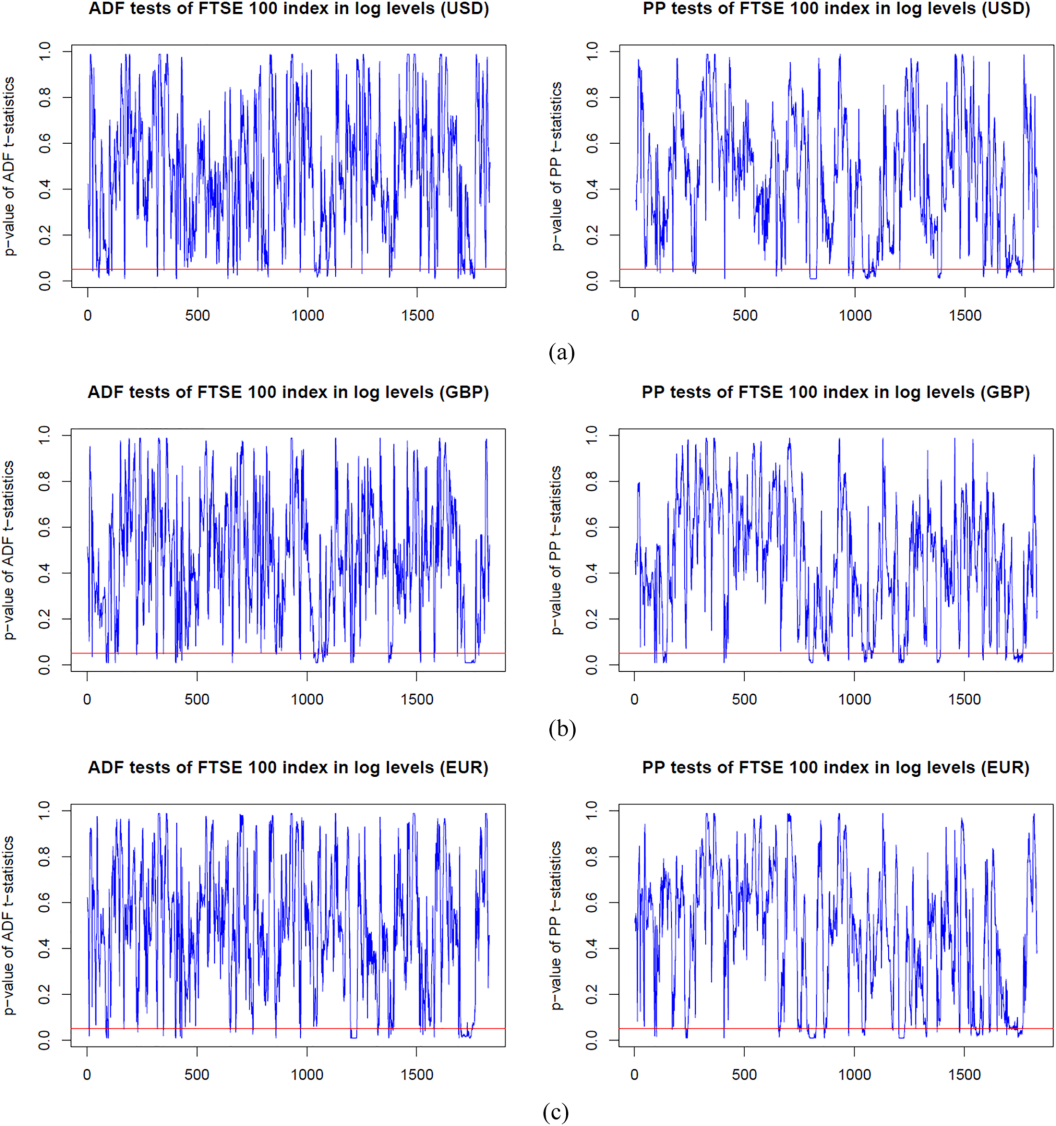
*p*-values from dynamic ADF and PP unit root tests of the FTSE 100 index based on USD, GBP and EUR respectively, in log levels. The red line indicates 5% statistical significance level. (a) FTSE 100 index in log level (USD). (b) FTSE 100 index in log level (GBP). (c) FTSE 100 index in log level (EUR).

**Fig 5:**
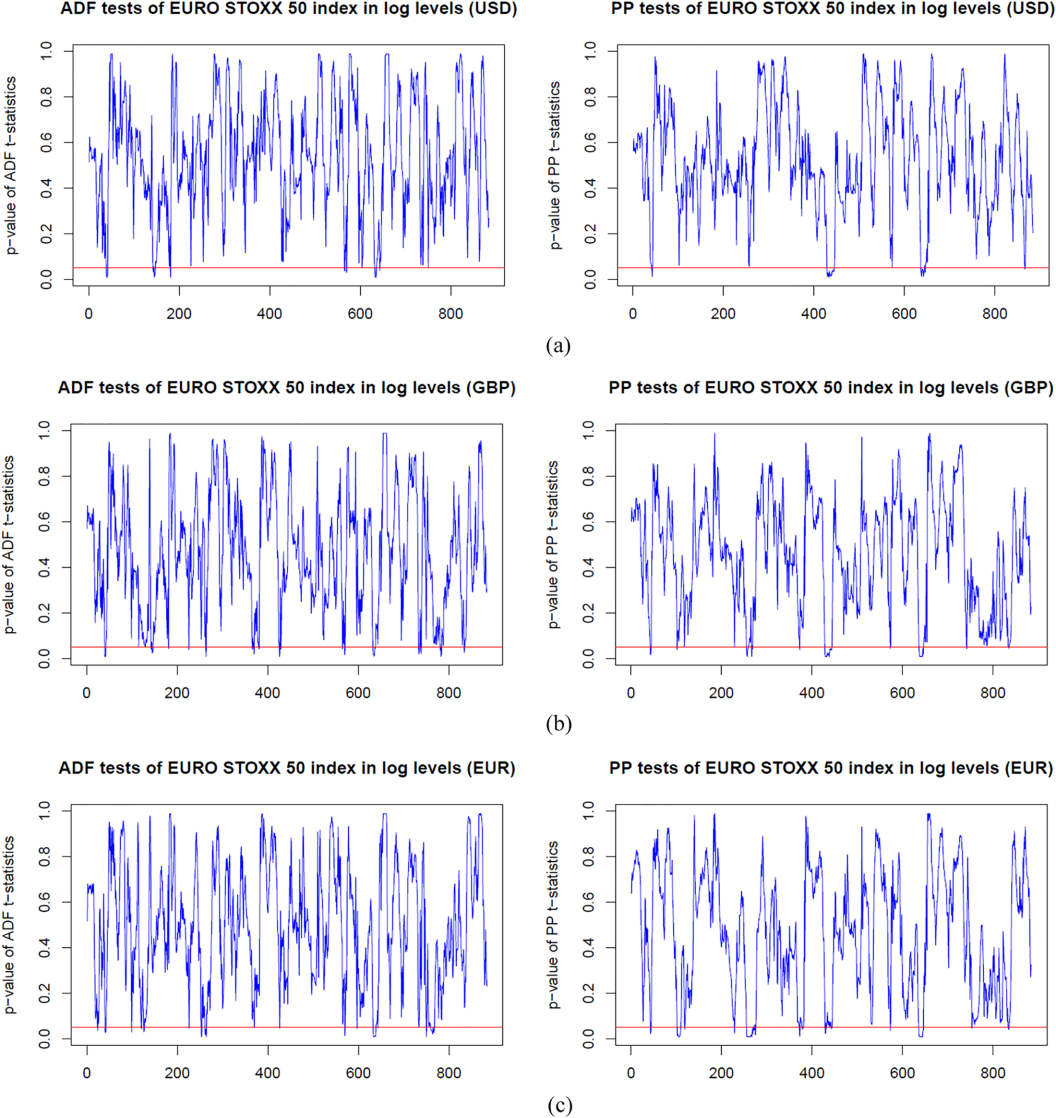
*p*-values from dynamic ADF and PP unit root tests of the EURO STOXX 50 index based on USD, GBP and EUR respectively, in log levels. The red line indicates 5% statistical significance level. (a) EURO STOXX 50 index in log level (USD). (b) EURO STOXX 50 index in log level (GBP). (c) EURO STOXX 50 index in log level (EUR).

Before estimating the dynamic cointegration in the long-run, we firstly employ the ADF and PP unit root tests models to examine the integration order of the S&P 500, FTSE 100 and EURO STOXX 50 indices in log levels and first differences. In Figs 3–5, we plot the dynamic p-values of ADF and PP *t*-statistic of the S&P 500, FTSE 100 and EURO STOXX 50 indices (expressed in USD, GBP and EUR, respectively) in logarithm levels. We observe that the *p*-values are above the red lines (5% significance level) for the vast majority of time windows. Thus, the null hypothesis of *γ* = 0 is accepted, and the stock indices are found to be non-stationary in log levels. However, for those cases in which the *p*-values are below the red lines, we have to delete the corresponding rolling windows to ensure that all stock index series under all sub-sample windows are *I*(1), i.e., non-stationary in logarithm levels and stationary in first differences. Since results imply that the stock index series contain a unit root in log levels and thus should be differenced to achieve stationarity. For the sake of space, we have not included the figures here. However, the dynamic *p*-values of ADF and PP *t*-statistic of the S&P 500, FTSE 100 and EURO STOXX 50 indices (expressed in USD, GBP and EUR, respectively) in first differences are all below the 5% significance level. The results of the rolling-window ADF and PP tests suggest that the S&P 500, FTSE 100 and EURO STOXX 50 indices are *I*(1) processes, and then we can implement the cointegration tests to examine whether there are long-run cointegration relations between the pairs of processes.

### Dynamic long-run cointegration analysis

**Fig 6:**
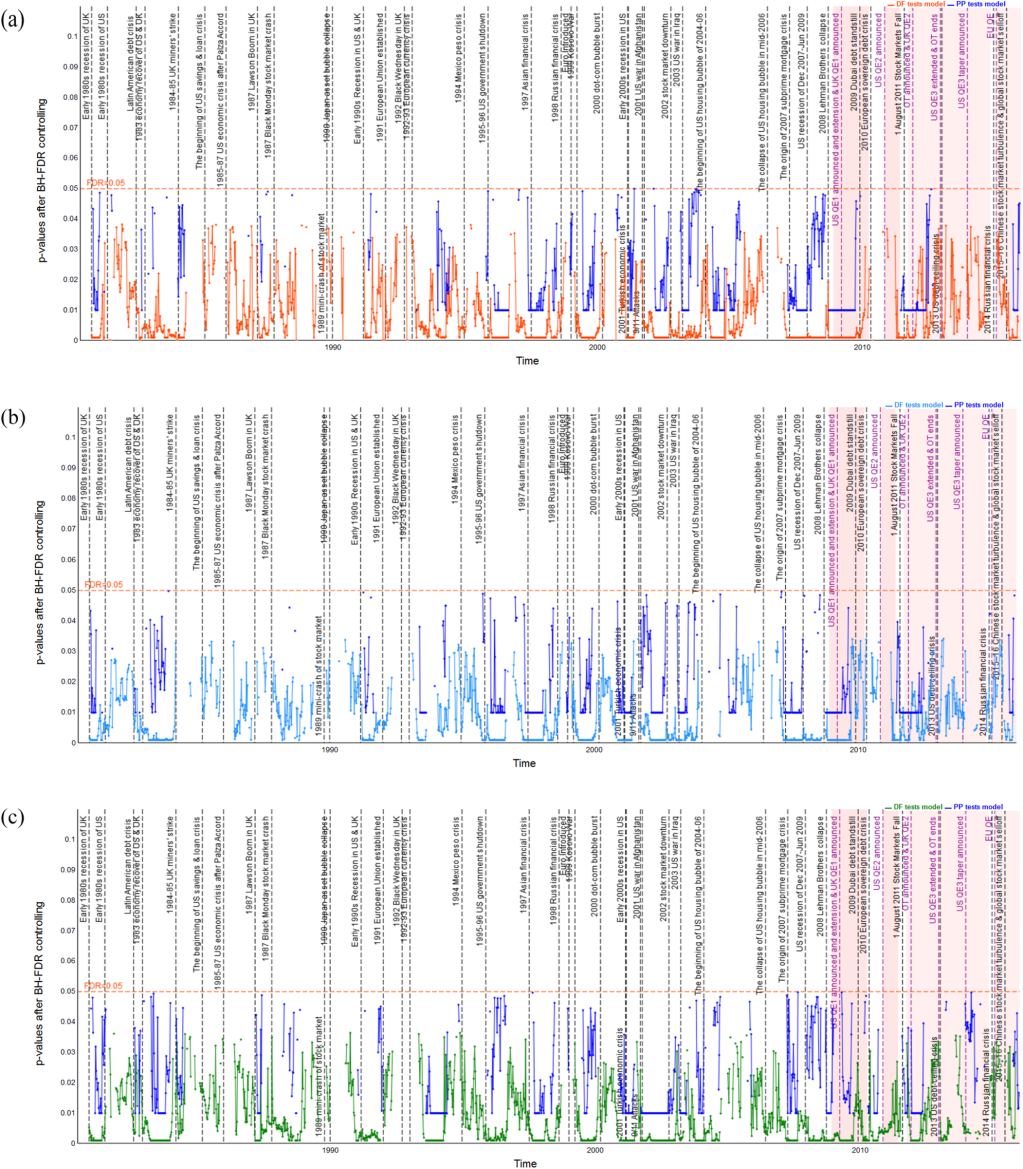
Dynamic *p*-values (based on DF and PP tests models) after BH FDR controlling showing FTSE 100’s cointegration with S&P 500 in USD, GBP and local currency terms, during 1980–2015. The red horizontal line denotes the false discovery rate with 0.05 for the multiple tests; black vertical lines correspond to economic, financial and political shocks during 1980–2015; red shading represents implementation of QE policies. (a) FTSE 100’s cointegration with S&P 500 measured in USD. (b) FTSE 100’s cointegration with S&P 500 measured in GBP. (c) FTSE 100’s cointegration with S&P 500 measured in local currencies.

**Fig 7:**
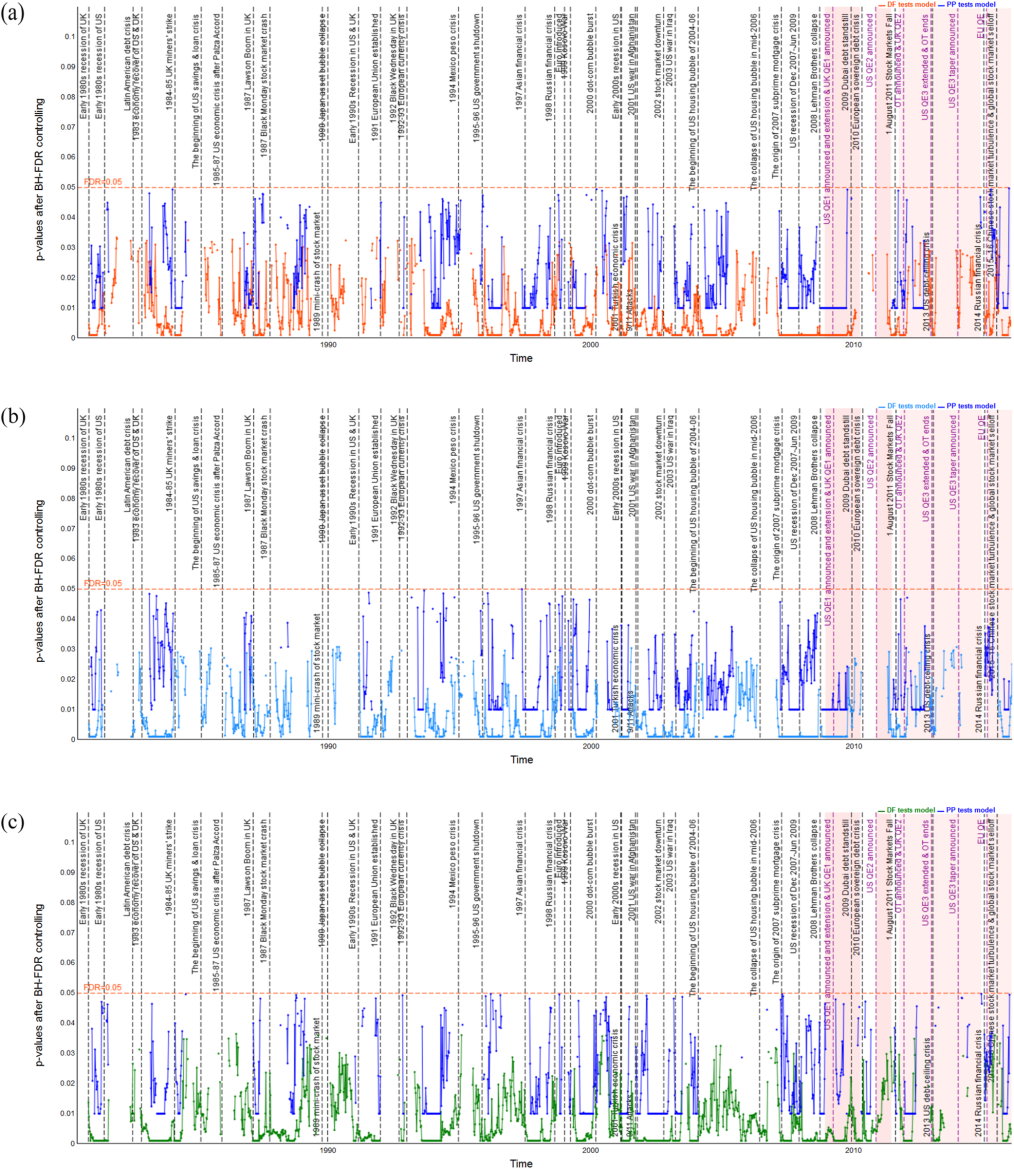
Dynamic *p*-values (based on DF and PP tests models) after BH FDR controlling showing S&P 500 100’s cointegration with FTSE 100 in USD, GBP and local currency terms, during 1980–2015. The red horizontal line denotes the false discovery rate with 0.05 for the multiple tests; black vertical lines correspond to external and internal economic, financial and political shocks during 1980–2015; red shading represents implementation of QE policies. (a) S&P 500’s cointegration with FTSE 100 measured in USD. (b) S&P 500’s cointegration with FTSE 100 measured in GBP. (c) S&P 500’s cointegration with FTSE 100 measured in local currencies.

**Fig 8:**
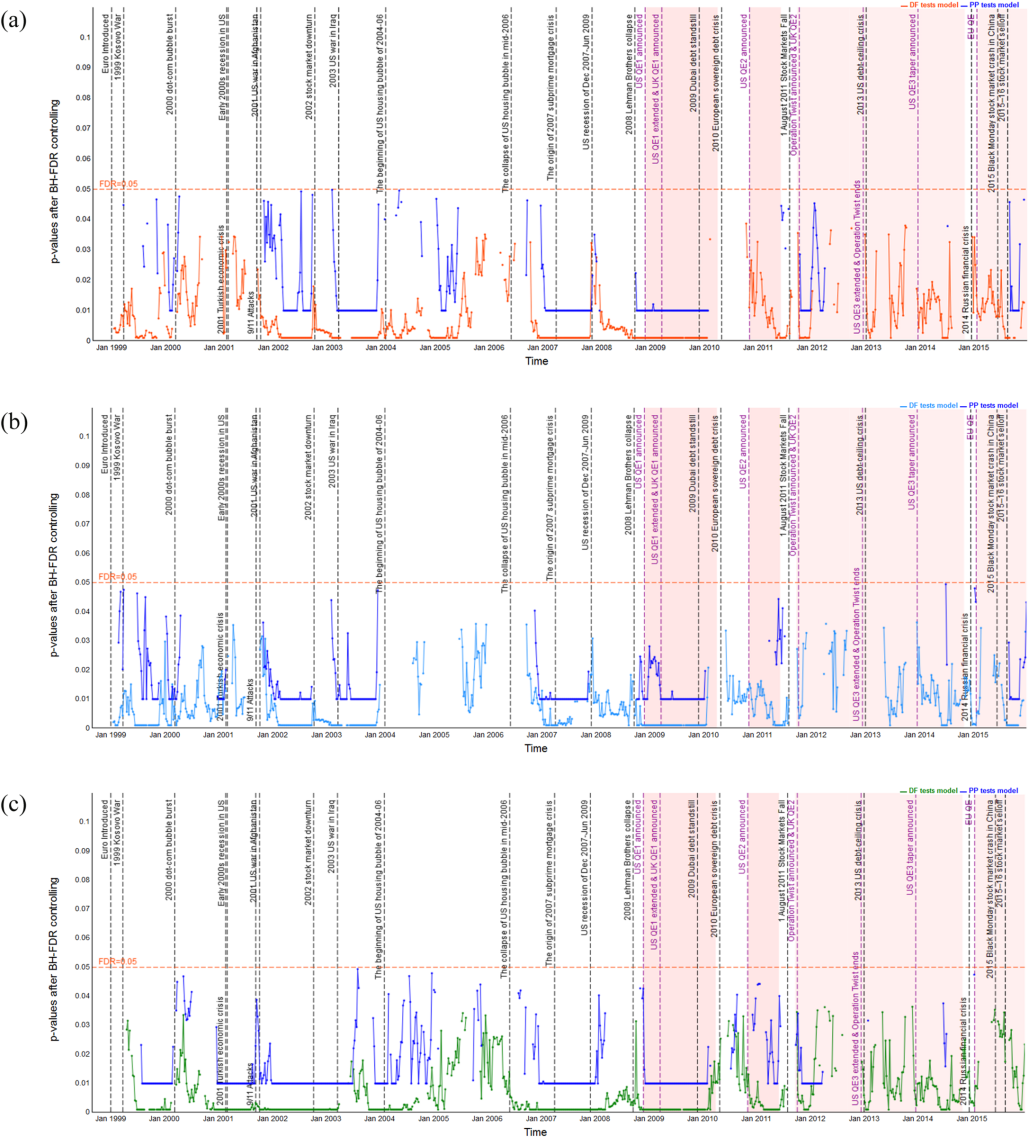
Dynamic *p*-values (based on DF and PP tests models) after BH FDR controlling showing EURO STOXX 50’s cointegration with S&P 500 in USD, EUR and local currency terms, during 1998–2015. The red horizontal line denotes the false discovery rate with 0.05 for the multiple tests; gray vertical lines correspond to external and internal financial shocks during 1998–2015; red shading represents implementation of QE policies.(a) EURO STOXX 50’s cointegration with S&P 500 measured in USD. (b) EURO STOXX 50’s cointegration with S&P 500 measured in GBP. (c) EURO STOXX 50’s cointegration with S&P 500 measured in local currencies.

**Fig 9:**
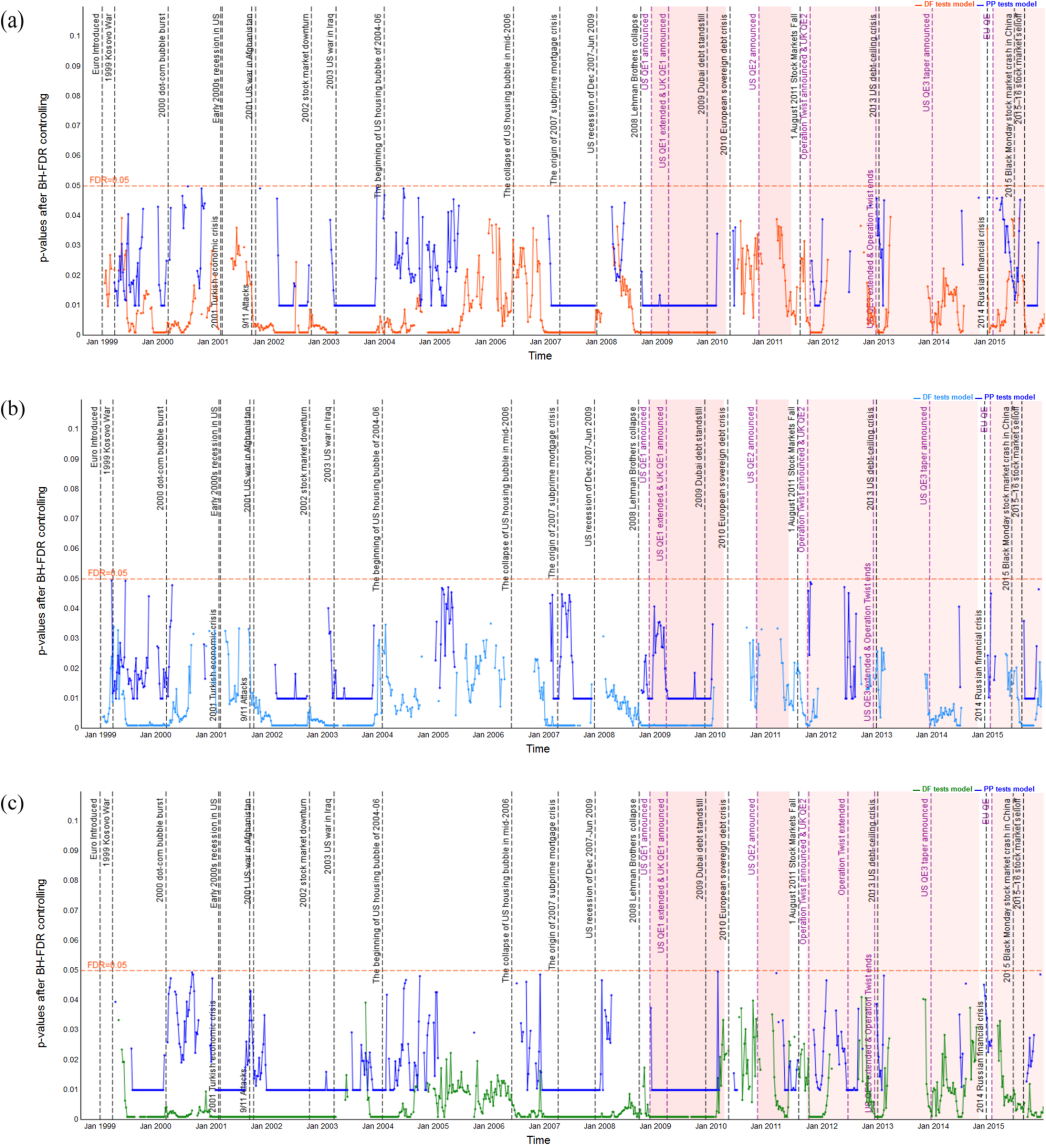
Dynamic *p*-values (based on DF and PP tests models) after BH FDR controlling showing S&P 500 100’s cointegration with EURO STOXX 50 in USD, EUR and local currency terms, during 1998–2015. The red horizontal line denotes the false discovery rate with 0.05 for the multiple tests; gray vertical lines correspond to external and internal financial shocks during 1998–2015; red shading represents implementation of QE policies.(a) S&P 500 100’s cointegration with EURO STOXX 50 measured in USD. (b) S&P 500 100’s cointegration with EURO STOXX 50 measured in GBP. (c) S&P 500 100’s cointegration with EURO STOXX 50 measured in local currencies.

**Fig 10:**
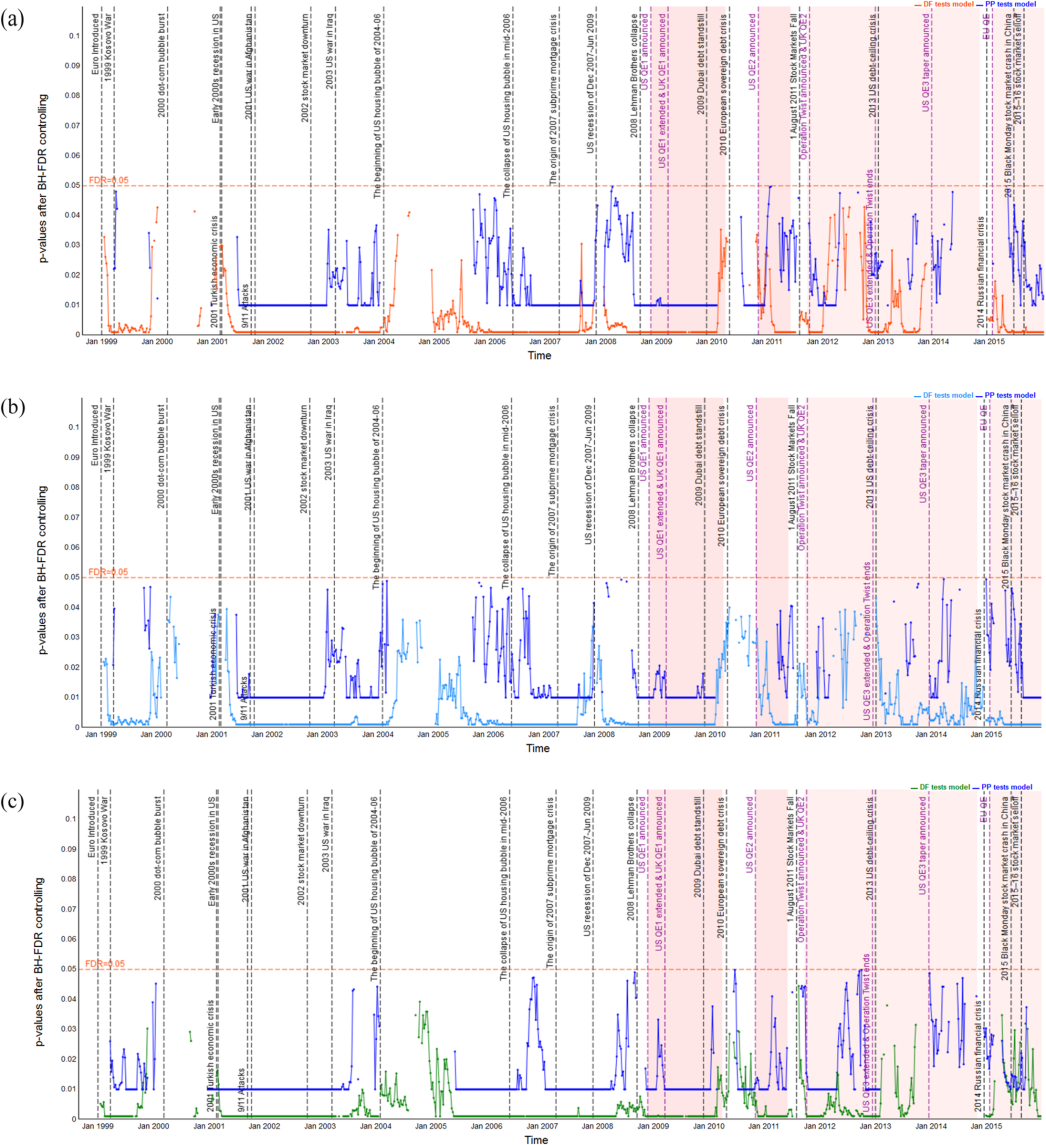
Dynamic *p*-values (based on DF and PP tests models) after BH FDR controlling showing EURO STOXX 50’s cointegration with FTSE 100 in GBP, EUR and local currency terms, during 1998–2015. The red horizontal line denotes the false discovery rate with 0.05 for the multiple tests; gray vertical lines correspond to external and internal financial shocks during 1998–2015; red shading represents implementation of QE policies.(a) EURO STOXX 50’s cointegration with FTSE 100 measured in GBP. (b) EURO STOXX 50’s cointegration with FTSE 100 measured in EUR. (c) EURO STOXX 50’s cointegration with FTSE 100 measured in local currencies.

**Fig 11:**
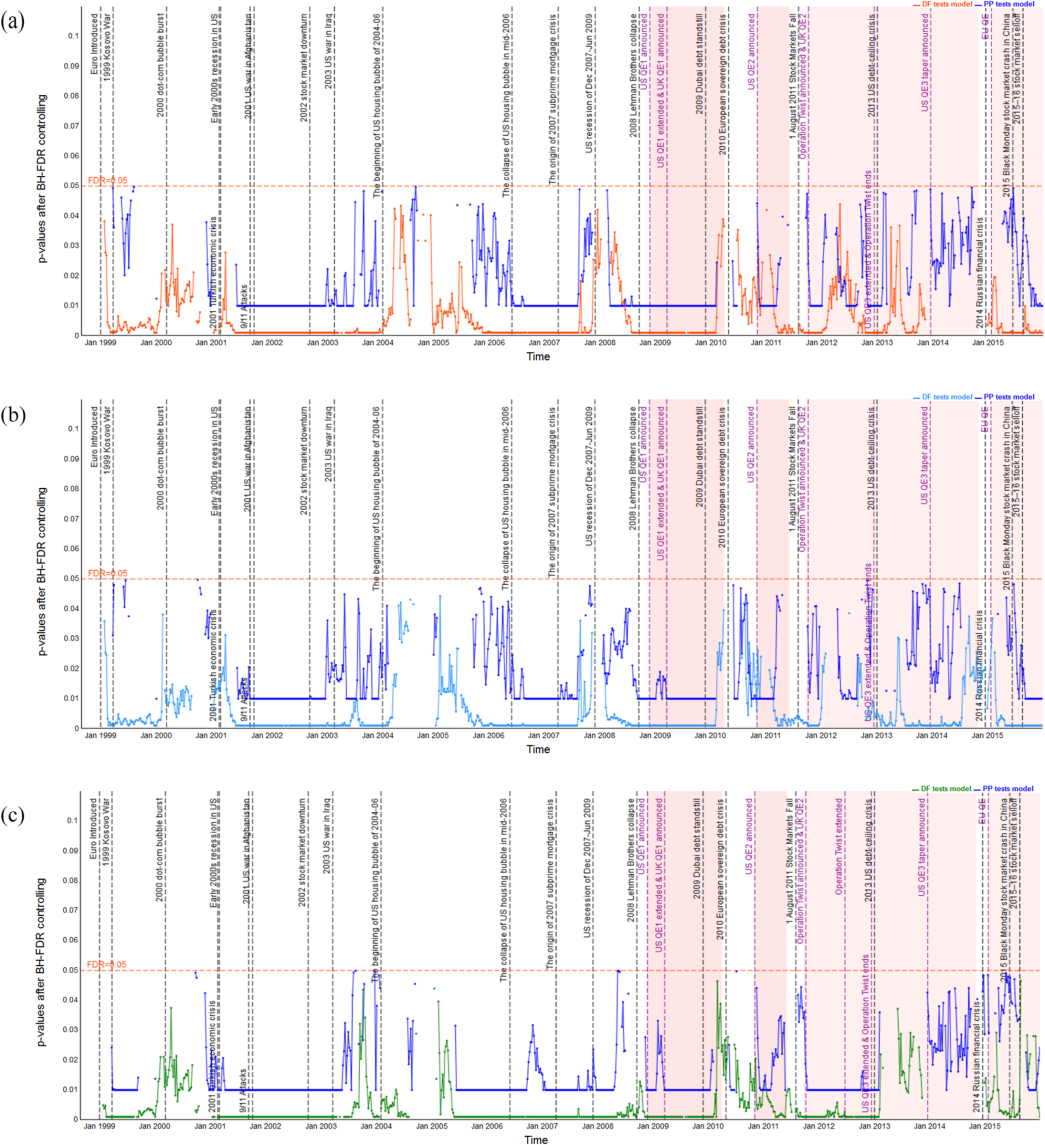
Dynamic *p*-values (based on DF and PP tests models) after BH FDR controlling showing FTSE 100’s cointegration with EURO STOXX 50 in GBP, EUR and local currency terms, during 1998–2015. The red horizontal line denotes the false discovery rate with 0.05 for the multiple tests; gray vertical lines correspond to external and internal financial shocks during 1998–2015; red shading represents implementation of QE policies.(a) FTSE 100’s cointegration with EURO STOXX 50 measured in GBP. (b) FTSE 100’s cointegration with EURO STOXX 50 measured in EUR. (c) FTSE 100’s cointegration with EURO STOXX 50 measured in local currencies.

Pairwise dynamic cointegration of stock indices is indicated by the *p*-values of the DF and PP unit root tests of the residual series; see Figs 6–11 which show the *p*-values after BH-FDR control for both *I*(1) process. In the multiple statistical tests, an FDR *p*-value that is consistently less than 0.05 or 0.01 would suggest that the null hypothesis of no cointegration could be rejected. Practically, this would mean that there is a long-run cointegration relationship between that pair of stock indices. Generally, the smaller the obtained *p*-values, the null hypothesis that there is no cointegration relationship can be rejected at lower values of the chosen statistical threshold. The one-year rolling cointegration estimation and the results based on DF and PP tests models for the dynamic *p*-values over the period 1980–2015 are plotted in Figs 6 and 7 for the S&P 500 and FTSE 100 measured in USD/USD, GBP/GBP, and their domestic currency units. Figs 8 and 9 show the S&P 500 and EURO STOXX 50 measured in USD/USD, EUR/EUR and their local currency units, and Figs 10 and 11 show the FTSE 100 and EURO STOXX 50 measured in GBP/GBP, EUR/EUR and their local currency units. Over 1980–2015, we can observe that the dynamic *p*-values vary over time, indicating significant fluctuation in the degree of integration among the different indices and currencies.

#### Dynamic cointegration between S&P 500 and FTSE 100 indices

The dynamic *p*-values that reflect the extent to which the FTSE 100 cointegrates with the S&P 500, measured in USD/USD, GBP/GBP and GBP/USD, are shown in Fig 6. Table 4 reports the observed time periods in which the FTSE 100 cointegrates with the S&P 500 at both the 1% and 5% significance levels based on both DF and PP tests of residuals. Combining the results of Fig 6 and Table 4, we find that the FTSE 100 cointegrates with the S&P 500 at the 1% significance level during the periods associated with the economic, financial and political shocks, from 1980 to 2015 based on both DF and PP tests. However, the results based on PP tests model are non-significant from 1980 to 1993. Based on the degree of persistent cointegration, an interesting finding is that when compared to the shocks that occurred in the developing countries (e.g., see periods 17, 19, 21), the shocks in the US market (e.g., see periods 18, 27, 29, 33–35) have a more significant influence on the FTSE 100’s cointegration behavior with the S&P 500. In particular, the most persistent periods of the FTSE 100’s cointegration with the S&P 500 are periods 33–35 based on both DF and PP tests, namely, the recent 2007–09 international financial crisis, which indicates that the US stock market significantly influenced the UK market during that time. On the other hand, the dynamic *p*-values exhibit lasting fluctuation during periods 2, 7, 31, 32, and 46–48, at the 5% statistical significance level. The observed results suggest that the 1985–87 US economic crisis caused by the Palza Accord [65], the continuous impact of the US housing asset bubble in 2004–06, and the US QE3 announced and taper policies implemented by the Federal Reserve [66] that are the most significant causes of the evidence of the FTSE 100’s cointegration with the S&P 500. Fig 6(a)–6(c) also illustrates the comparative analysis of how exchange rate movements affect the cointegration of the FTSE 100 with the S&P 500 based on both DF and PP tests. At first sight, the difference between the cointegration as measured in the same currencies versus local currencies seems relatively small, while in periods 9, 39–40 and 49 we can observe stronger integration when measured in local currency terms, GBP/USD, which is in line with the findings of Voronkova’s study [42]. During period 24, the evidence that the FTSE 100 cointegrates with the S&P 500 can only be found when measured using local currencies, which is consistent with Alexander [41]. Furthermore, there is a stronger possibility that the FTSE 100 cointegrates with the S&P 500 when we measure it using USD/USD and domestic currency terms during periods 5, 16 and 31. On the contrary, the evidence of cointegration disappears when we measure it using GBP/GBP (note that the GBP depreciated against the USD during these periods). Reverse findings are identified during periods 8 and 40. In these periods, the evidence of the FTSE 100’s cointegration with the S&P 500 vanishes when measured in USD/USD (the USD depreciated against the GBP during these periods).

**Table 4:** Observed periods of cointegration and Granger causality (in long run) between the S&P 500 and FTSE 100 during 1980–2015.

S&P 500 vs. FTSE 100	Observed periods		
**S&P 500 → FTSE 100**	USD/USD	GBP/GBP	GBP/USD
At 1% significance level	periods 1, 3–5, 8–10	periods 1, 3, 4, 9	periods 1, 3–5, 8, 9
	periods 13, 14, 16, 18–20	periods 13–14,16, 18–20	periods 13, 16, 18–20
	periods 23, 25–31, 33–38	periods 23, 25–30, 33–38	periods 23, 25–31, 33–44
	periods 41–44, 49, 52–53	periods 42–44, 49–50, 52–53	periods 46–48, 52–53
At 5% significance level	periods 2, 6, 7	periods 2, 6–8, 10–12	periods 2, 6, 7, 10–12
	periods 11, 17, 24, 32	periods 17, 24, 31, 32	periods 14, 17, 24, 32
	periods 39, 40, 46–48, 50, 51	periods 39, 40, 45–48, 51	periods 45, 50–51
**S&P 500 causes FTSE 100**	periods 1–10, 13–14, 18, 22–23	periods 1–2, 7, 9, 12–14, 16–17	periods 1–2, 9, 12–14, 17
	periods 27–29, 31, 33–34	periods 22–24, 26–27, 29, 31–34	periods 19–24, 26, 31–38
	periods 42, 46–49, 52–53	periods 38, 41, 44, 46–51	periods 45–51
(53 sub-periods)	53%	53%	55%
**FTSE 100 → S&P 500**	USD/USD	GBP/GBP	USD/GBP
At 1% significance level	periods 1, 4, 5, 8	periods 1, 4, 7–9	periods 1, 4, 5, 8, 9
	periods 16–21, 23, 25–31	periods 13, 16, 18–20, 23	periods 13, 15, 16, 18–21, 23
	periods 33–38, 41–44	periods 25–30, 33–38	periods 25–30, 32–40, 42, 44
	periods 49–53	periods 41–44, 49, 52–53	periods 49, 52–53
At 5% significance level	periods 2, 3, 6, 7, 9, 11, 13–15	periods 2, 5, 6, 11, 14, 31	periods 3, 7, 11, 12, 14, 24
	periods 24, 32, 39–40, 45–48	periods 39, 46–48	periods 31, 41, 50–51
**FTSE 100 causes S&P 500**	periods 1–2, 5, 7, 9, 11	periods 1, 3–5, 7–8, 11, 13	periods 1, 3–5, 11, 15–16
	periods 15–17, 19–21, 24–25	periods 15–16, 18–19, 21, 24, 26	periods 18, 24, 27–31, 40
	periods 30–31, 35, 40, 50–53	periods 28, 30–31, 35–38	periods 42, 52–53
(53 sub-periods)	42%	42%	34%

Note that, to indicate that *A* cointegrates with *B*, we write *B* → *A*.

Fig 7 shows the dynamic *p*-values that indicate the S&P 500’s cointegration with the FTSE 100, measured in USD/USD, GBP/GBP, and USD/GBP, at both 1% and 5% significance levels based on both DF and PP tests models of residuals. Similarly, Table 4 reports the observed times at which the S&P 500 cointegrates with the FTSE 100, all of which are associated with economic, financial and political episodes that occurred during 1980–2015. The most long-lasting period of cointegration occurs during periods 33–35, i.e., during the 2007–09 global financial crisis, which was also the case for the FTSE 100’s cointegration with the S&P 500. However, when comparing Figs 6 and 7, one difference we can see is that the dynamic *p*-values are greater for the S&P 500 cointegrating with the FTSE 100 than vice versa, which suggests a lower degree of cointegration. In particular, during period 2, the time of the early-1980s recession in the US market, the evidence of the S&P 500 cointegrating with the FTSE 100 disappears. Furthermore, during periods 46–48, when the US QE3 and tapering policies announced, we find evidence that the S&P 500’s long-lasting cointegration with the FTSE 100 is weak and almost disappears. Additionally, the evidence indicates that, since the growth of the FTSE 100 lagged significantly behind that of the S&P 500, following the severe shocks caused by the 2007–09 global financial crisis and 2010 European sovereign debt crisis, the influence of the UK on the US market was weaker than the reverse. In contrast, the degree of the S&P 500’s cointegration with the FTSE 100 tends to be higher than that of the FTSE 100’s cointegration with the S&P 500 during period 17, namely, the 1994 Mexican debt crisis. Moreover, we notice that the S&P 500 cointegrating with the FTSE 100 significantly during period 15 (i.e., 1992’s “Black Wednesday” in the UK), while the FTSE 100 does not cointegrate with the S&P 500 during that period (see Fig 6). These results imply that the UK currency crisis on September 16th, 1992 not only affected the UK stock market greatly but also enhanced the latter’s influence on the US market.

Finally, taking into account the influence of exchange rate movements on the S&P 500’s dynamic long-lasting cointegration with the FTSE 100 based on both DF and PP tests results (see Fig 7(a)–7(c)), we observe that, during periods 5, 9, 31 and 39, the S&P 500 cointegrates more intensely with the FTSE 100 when they are measured in USD/USD and local currency terms, respectively. In particular, the S&P 500’s cointegration with the FTSE 100 can only be identified when using the local currencies during period 40, namely during the 2010 European sovereign debt crisis. Furthermore, our results reveal that, during periods 15, 50–51, the evidence that the S&P 500 cointegrates with the FTSE 100 disappears when measured in GBP/GBP (note that there was depreciation of the GBP against the USD during these periods), while it is stronger when measured in USD/USD and local currency terms. The opposite results are observed during period 13, when a higher degree of cointegration is reported under GBP/GBP and the local currencies, yet there is no evidence of cointegration under USD/USD (note the depreciation of the USD against the GBP at this time).

#### Dynamic cointegration between the S&P 500 and EURO STOXX 50 indices

The dynamic *p*-values based on DF and PP tests of residuals indicating the extent to which the EURO STOXX 50 cointegrates with the S&P 500 and the S&P 500 cointegrates with EURO STOXX 5 under common and local currency terms, are only presented from 1998 to 2015 (see Figs 8 and 9). Table 5 gives the observed periods of cointegration between stock markets of the US and Eurozone for both the 1% and 5% statistical significance levels. From Figs 8 and 9, we can observe similar degrees of long-lasting cointegration of the EURO STOXX 50 with the S&P 500 and vice versa, associated with economic and financial shocks, and once again the cointegration between the S&P 500 and EURO STOXX 50 is most persistent and highest during the 2007–09 global financial crisis, out of the whole sample period. However, a significant distinction is that, during periods 24 and 31, namely after the 2000 bursting of the dot-com bubble and during the 2004–06 US housing asset bubble, there is stronger cointegration of the S&P 500 with the EURO STOXX 50 than vice versa. However, the opposite is true for periods 46–48, i.e., when the US QE3 and tapering policies were implemented.

**Table 5:** The observed periods of cointegration and Granger causality (in long run) between the S&P 500 and EURO STOXX 50, during 1998–2015.

S&P 500 vs. EURO STOXX 50	Observed periods		
**S&P 500 → EURO STOXX 50**	USD/USD	EUR/EUR	EUR/USD
At 1% significance level	periods 22, 23, 27–39	periods 22–24, 27–30	periods 23–29, 31–39
	periods 41–44	periods 32–39,	periods 41–44, 46–50
	periods 49, 52–53	periods 41, 52–53	periods 52–53
At 5% significance level	periods 24–26, 46–48	periods 25, 26, 31, 40	periods 30, 40, 45
	periods 50–51	periods 42–48, 50–51	periods 51
**S&P 500 causes EURO STOXX 50**	periods 22–23, 27–34	periods 22–23, 25	periods 22–23, 25
	periods 38–39, 41	periods 27–34, 40–41	periods 27–36, 38–39, 41
	periods 49–53	period 49–51	periods 50–51
(32 sub-periods)	(56%)	(50%)	(56%)
**EURO STOXX 50 → S&P 500**	USD/USD	EUR/EUR	USD/EUR
At 1% significance level	periods 23, 24, 27–31, 33	periods 22–24, 27–30	periods 23–39
	periods 35–39, 42, 44	periods 33, 35–39, 44	periods 41, 43–49
	periods 46–53	periods 46–48, 52–53	periods 50–51
At 5% significance level	periods 22, 25, 26, 32	periods 25, 26, 31, 34	periods 40, 42
	periods 34, 40, 41	periods 40, 41, 49–51	
**EURO STOXX 50 causes S&P 500**	periods 24–25,27–28, 31	periods 24–25, 27–28, 31	periods 24–28, 31
	periods 35–36, 40, 42–44	periods 35–36, 38–39	periods 40, 42–44
	periods 46–51	periods 42–44, 46–49	periods 46–49, 52–53
(32 sub-periods)	(53%)	(50%)	(50%)

Note that, to indicate that *A* cointegrates with *B*, we write *B* → *A*.

Now turning our attention to how changes in exchange rates influence the integration behavior between the S&P 500 and EURO STOXX 50, we compare Figs 8(a)–8(c) and 9(a)–9(c). Based on the results of both DF and PP tests models, there is a stronger probability of the existence of cointegration between the S&P 500 and EURO STOXX 50 when they are measured in their local currencies rather than under a common currency, i.e., USD/USD and EUR/EUR, respectively. Particularly, during periods 26 and 27, there is a larger probability of cointegration between the EURO STOXX 50 and S&P 500 when they are measured in local currency terms. Furthermore, the EURO STOXX 50 appears to cointegrate more strongly with the S&P 500 during periods 31 and 44 when they are measured in USD/USD and local currency terms, yet the evidence of cointegration is weaker under EUR/EUR (note the depreciation of the EUR against the USD during these periods). Besides, Fig 8 reveals that during period 40, the evidence that the EURO STOXX 50 cointegrates with the S&P 500 is significant only when it is measured in EUR/EUR and the local currencies, while no cointegration appears under USD/USD. On the other hand, as for the evidence of the S&P 500 cointegrating with the EURO STOXX 50, during periods 26, 31, 34, 41, 45, 46–48 and 51, we observe stronger cointegration when they are measured in local currency terms.

#### Dynamic cointegration between the FTSE 100 and EURO STOXX 50 indices

Figs 10 and 11 show the dynamic *p*-values based on both DF and PP tests of residuals indicating the extent to which the EURO STOXX 50 cointegrates with the FTSE 100 and vice versa, measured in both common and local currency terms, for 1998–2015. Table 6 shows all the periods of integration at both 1% and 5% statistical significance levels. Table 6 reports that the periods during which the EURO STOXX 50 cointegrates with the FTSE 100 and the FTSE 100 cointegrates with the EURO STOXX 50 are quite similar during the whole sample period. In particular, for periods 31–39, there is the strongest probability of cointegration existing between the FTSE 100 and EURO STOXX 50, out of the entire sample period. We also observe that the FTSE 100 cointegrates with the EURO STOXX 50 only during periods 24 and 40, while there is no evidence that the EURO STOXX 50 cointegrates with the FTSE 100. The reason might be related to the severe debt crisis in the Eurozone, which led to more shocks moving from the Eurozone to the UK stock market than vice versa. In addition, since the EURO STOXX 50 index covers 50 stocks from 11 Eurozone countries (i.e., Austria, Belgium, Finland, France, Germany, Ireland, Italy, Luxembourg, the Netherlands, Portugal and Spain), it appears that the collapse of the dot-com asset bubble in the US in March 2000 affected the EURO STOXX 50 more than the FTSE 100 index.

**Table 6:** The observed periods of cointegration and Granger causality (in long run) between the FTSE 100 and EURO STOXX 50 during 1998–2015.

FTSE 100 vs. EURO STOXX 50	Observed periods		
**FTSE 100 → EURO STOXX 50**	GBP/GBP	EUR/EUR	EUR/GBP
At 1% significance level	periods 22, 23, 26–39	periods 22, 23, 26–39	periods 22, 23, 26–39
	periods 41–44, 46–53	periods 41–44, 46–53	periods 41–48, 52–53
At 5% significance level	period 40	period 40	periods 40, 50–51
**FTSE 100 causes EURO STOXX 50**	periods 22–23, 29–31, 33	periods 22, 29–30, 33–34	periods 22–23, 29–31
	periods 35–36, 39, 41	periods 40–41	periods 33–34, 36, 41
	periods 50–51, 52–53	periods 50–53	periods 50–53
(32 sub-periods)	44%	34%	47%
**EURO STOXX 50 → FTSE 100**	GBP/GBP	EUR/EUR	GBP/EUR
At 1% significance level	periods 22, 23, 25–39	periods 22, 23, 25–39	periods 22, 23, 25–39
	periods 41, 44–53	periods 41, 44–53	periods 41–46, 49–53
At 5% significance level	periods 24, 40, 42	periods 24, 40, 42	periods 24, 40, 48
**EURO STOXX 50 causes FTSE 100**	periods 24, 26–28, 34	periods 27–28, 36	periods 24–28, 31
	periods 40, 44	periods 40, 44	periods 43, 45–46
	periods 46–48	periods 46–48	periods 50–51
(32 sub-periods)	31%	25%	34%

Note that, to indicate that *A* cointegrates with *B*, we write *B* → *A*.

Regarding the influence of exchange rate movements, Table 6 reports the cointegration between the FTSE 100 and the EURO STOXX 50. Of particular note, during periods 40 and 45, we identify stronger cointegration of the EURO STOXX 50 with the FTSE 100 and vice versa when using the local currencies. Furthermore, during periods 46–48, i.e., the US Fed implemented the QE3 and tapering policies, there is strong persistent cointegration of the FTSE 100 and EURO STOXX 50. These results indicate that the economic recession in the UK and Eurozone markets and a series of similar monetary and fiscal policies caused these two markets to integrate significantly.

To sum up, based on the dynamic cointegration analysis between all pairs of stock market indices, we conclude that the persistent cointegration periods observed are associated with asset bubbles, market crashes, sovereign failures, or wars. In particular, during the 2007–09 global financial crisis, all three major stock markets exhibited the most persistent and deepest cointegration with each other due to the serious shocks on the US and global stock markets based on both DF and PP tests. There is some evidence that, during economic, financial and political shocks, the capitalization of the stock market indices grew quickly and synchronously, and they were highly cointegrated with each other. Meanwhile, when an individual stock market experiences economic, financial and political episodes (e.g., see the 2004–06 US housing asset bubble, the 2010 European sovereign debt crisis, etc.), it is significantly affected by other stock markets due to the recession in the former country’s economy. Furthermore, by comparing with the dynamic correlation between S&P 500 and FTSE 100, S&P 500 and EURO STOXX 50, FTSE 100 and EURO STOXX 50, the degree of cointegration changed associated with the rising or decreasing correlation obviously. Additionally, when the indices are measured in local currency terms, the probability of cointegration between all three pairs of stock indices is higher than that when using the same unit of currency for each index in the pair, which is consistent with the findings of Voronkova’s study [42]. Evidence of cointegration can only be found when using local currencies during some time periods, which is in line with Alexander and Thillainathan [41], who also found that integration between international equity markets appeared only when stock indices were expressed in local currency terms. Our comparative analysis conducted under common and local currency terms, formulated on a dynamic framework, provides new insights over and above that found in the existing studies.

### Dynamic ECM-based long-run Granger causality analysis

**Fig 12:**
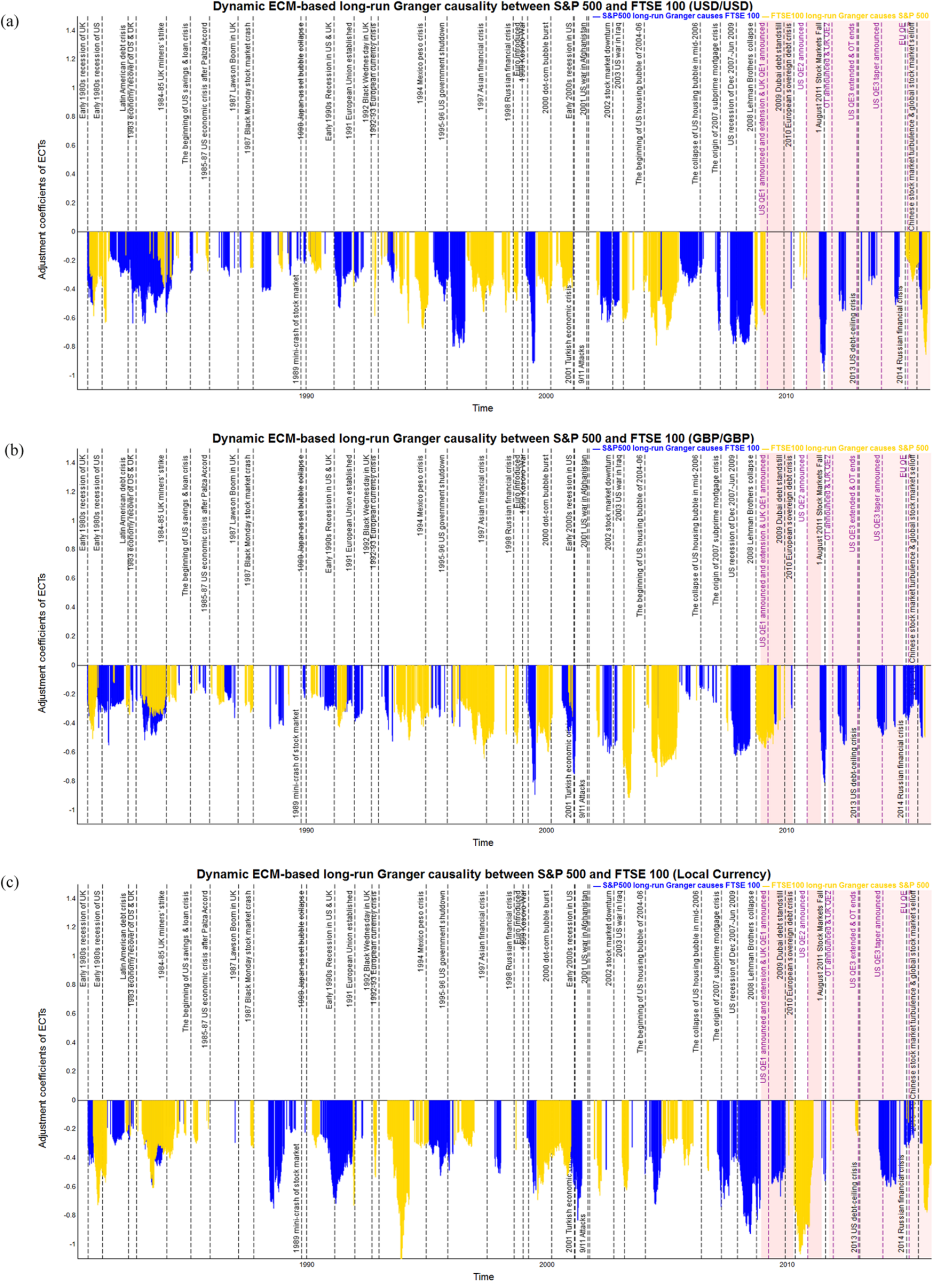
The statistical significant and negative dynamic ECM-based long-run Granger causality of S&P 500 and FTSE 100 measured in common and local currency terms in 1980–2015. The blue bars show the S&P 500 causes FTSE 100, and the yellow bars show the FTSE 100 causes S&P 500, respectively. The red shading represents implementation of QE policies. (a) Dynamic long-run Granger causality between S&P 500 and FTSE 100 measured in USD. (b) Dynamic long-run Granger causality between S&P 500 and FTSE 100 measured in GBP. (c) Dynamic long-run Granger causality between S&P 500 and FTSE 100 measured in local currencies.

**Fig 13:**
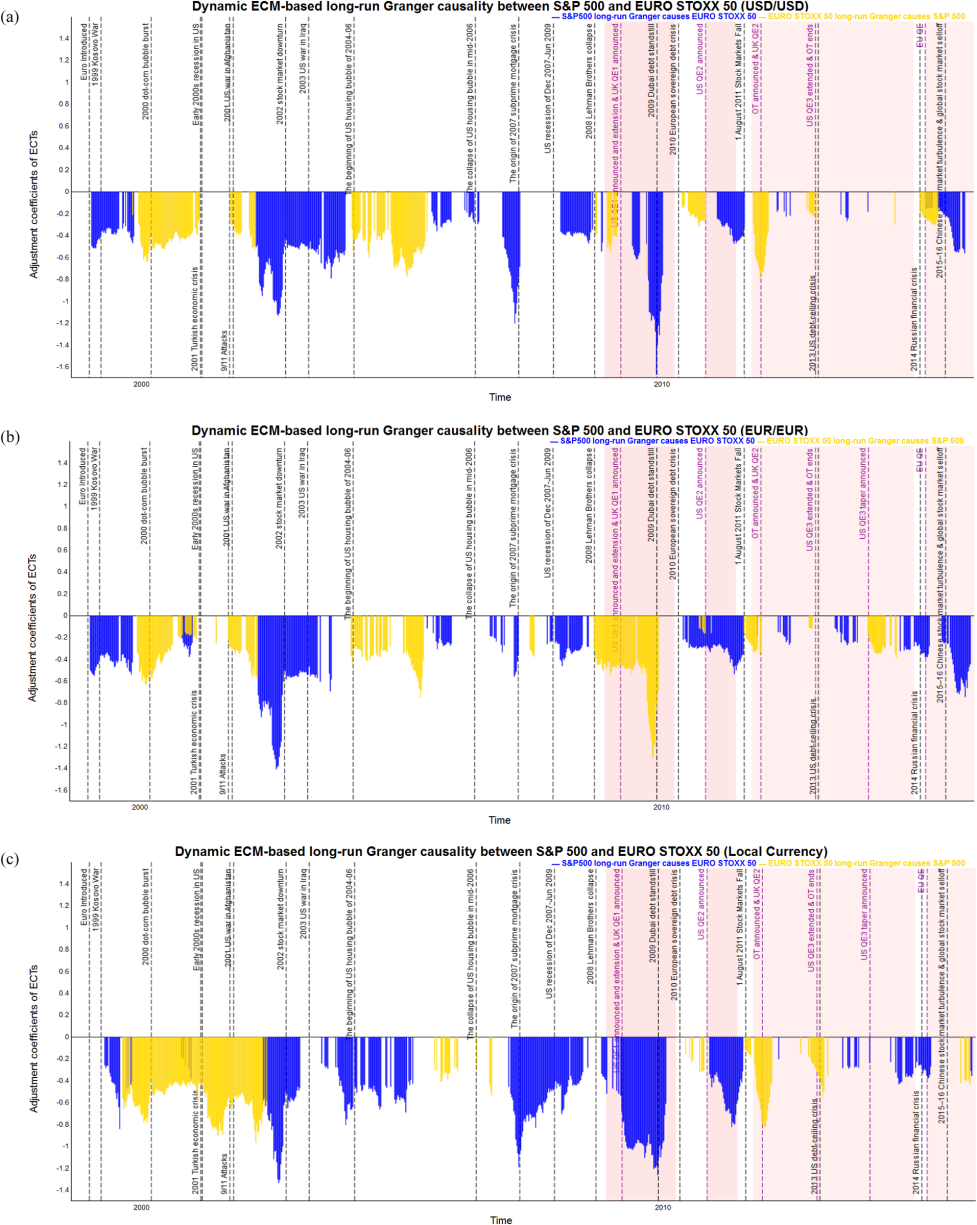
The statistical significant and negative dynamic ECM-based long-run Granger causality of S&P 500 and EURO STOXX 50 measured in common and local currency terms in 1998–2015. The blue bars show the S&P 500 causes EURO STOXX 50, and the yellow bars show the EURO STOXX 50 causes S&P 500, respectively. The red shading represents implementation of QE policies. (a) Dynamic long-run Granger causality between S&P 500 and EURO STOXX 50 measured in USD. (b) Dynamic long-run Granger causality between S&P 500 and EURO STOXX 50 measured in EUR. (c) Dynamic long-run Granger causality between S&P 500 and EURO STOXX 50 measured in local currencies.

**Fig 14:**
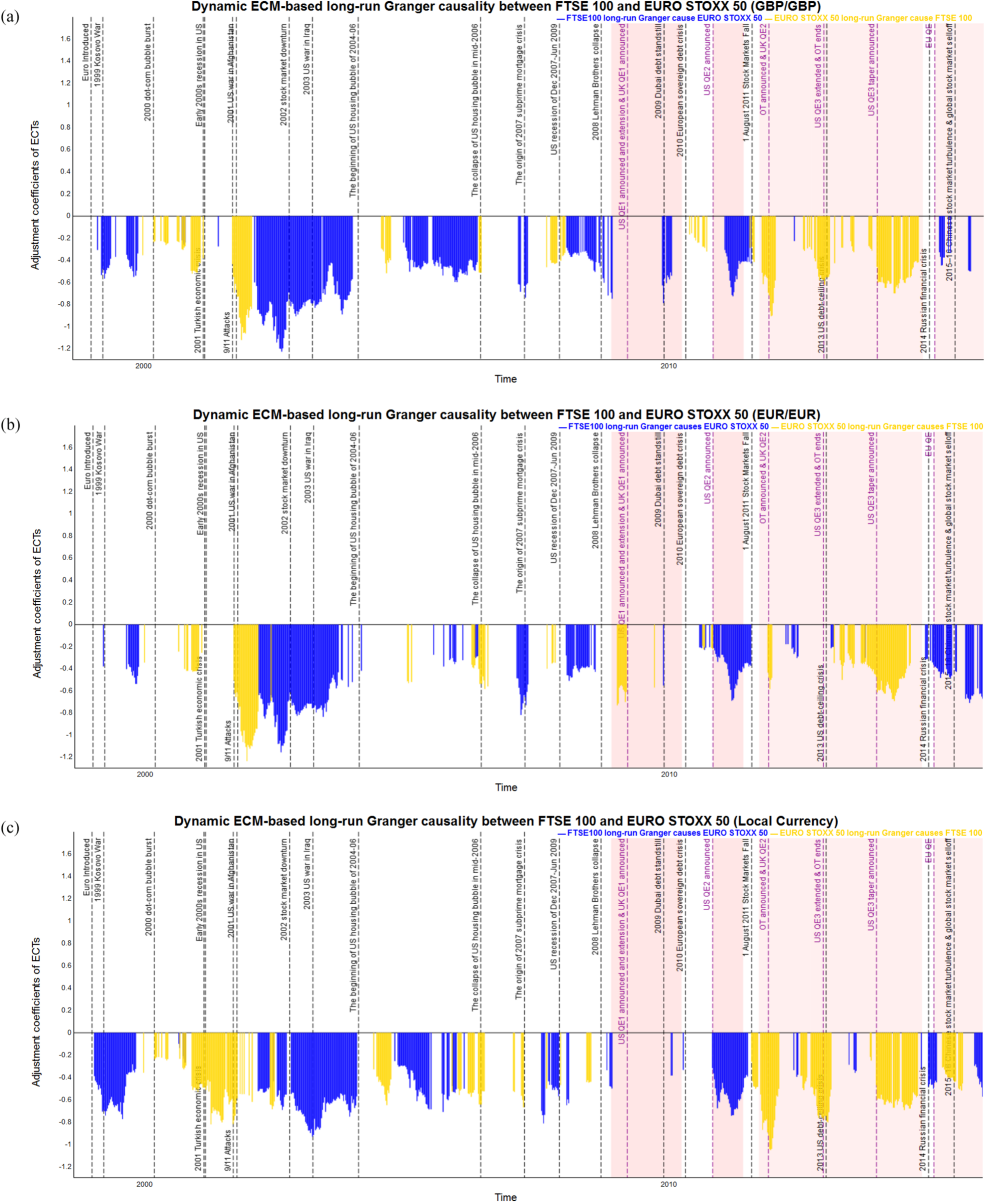
The statistical significant and negative dynamic ECM-based long-run Granger causality of FTSE 100 and EURO STOXX 50 measured in common and local currency terms in 1998–2015. The blue bars show the FTSE 100 causes EURO STOXX 50, and the yellow bars show the EURO STOXX 50 causes FTSE 100, respectively. The red shading represents implementation of QE. (a) Dynamic long-run Granger causality between FTSE 100 and EURO STOXX 50 measured in GBP. (b) Dynamic long-run Granger causality between FTSE 100 and EURO STOXX 50 measured in EUR. (c) Dynamic long-run Granger causality between FTSE 100 and EURO STOXX 50 measured in local currencies.

As was described in the previous subsection, the dynamic *p*-values based on DF and PP tests after BH-FDR controlling indicate the probability that we can accept the long-run cointegration between the pairs of stock market indices. Then, the ECM is used to identify the long-run Granger causality through the error correction coefficients. Only statistical significant error correction coefficients are reported in Figs 12 to 14 for each pair of stock market indices of S&P 500, FTSE 100 and EURO STOXX 50 from 1980 to 2015, respectively. In particular, Tables 4–6 report the time periods in which we observe the statistical significantly directional Granger causality between each pair of stock indices in the long run during 1980–2015. Table 7 provides the summary statistics in the form of strongest, weakest, and average absolute value of adjustment coefficients.

**Table 7:** Statistical analysis of dynamic error correction coefficients (absolute value) of ECTs.

Stock Market Indices	Strongest Coeff	Weakest Coeff	Average Coeff
**S&P 500 vs. FTSE 100**			
S&P 500 causes FTSE 100 (USD/USD)	0.970	0.124	0.387
FTSE 100 causes S&P 500 (USD/USD)	0.856	0.120	0.366
S&P 500 causes FTSE 100 (GBP/GBP)	0.895	0.116	0.336
FTSE 100 causes S&P 500 (GBP/GBP)	0.917	0.113	0.349
S&P 500 causes FTSE 100 (GBP/USD)	0.926	0.136	0.429
FTSE 100 causes S&P 500 (USD/GBP)	1.284	0.141	0.377
**S&P 500 vs. EURO STOXX 50**			
S&P 500 causes EURO STOXX 50 (USD/USD)	1.668	0.141	0.441
EURO STOXX 50 causes S&P 500 (USD/USD)	0.783	0.157	0.416
S&P 500 causes EURO STOXX 50 (EUR/EUR)	1.407	0.115	0.339
EURO STOXX 50 causes S&P 500 (EUR/EUR)	1.308	0.109	0.368
S&P 500 causes EURO STOXX 50 (EUR/USD)	1.332	0.191	0.504
EURO STOXX 50 causes S&P 500 (USD/EUR)	0.963	0.122	0.511
**FTSE 100 vs. EURO STOXX 50**			
FTSE 100 causes EURO STOXX 50 (GBP/GBP)	1.225	0.143	0.498
EURO STOXX 50 causes FTSE 100 (GBP/GBP)	1.119	0.154	0.425
FTSE 100 causes EURO STOXX 50 (EUR/EUR)	1.154	0.177	0.479
EURO STOXX 50 causes FTSE 100 (EUR/EUR)	1.233	0.191	0.504
FTSE 100 causes EURO STOXX 50 (EUR/GBP)	0.930	0.091	0.553
EURO STOXX 50 causes FTSE 100 (GBP/EUR)	1.050	0.215	0.524

In the case of the long-run Granger causality between S&P 500 and FTSE 100, Fig 12(a)–12(c) show the dynamic statistical significant error correction coefficients based on the results of the FTSE 100’s cointegration with the S&P 500 and the S&P 500 cointegration with the FTSE 100, calculated using the same and local currencies, respectively. We observe that all the adjustment coefficients for the ECTs are negative for S&P 500 and FTSE 100, confirming the long-run Granger causality running from S&P 500 towards FTSE 100 (shown with a blue bar), from FTSE 100 to S&P 500 (shown with a yellow bar), respectively. As shown in Table 4, the proportion of period in which the S&P 500 long-run Granger causes FTSE 100 is greater than the reverse, namely 53% to 42% when using USD/USD, 53% to 42% when using GBP/GBP, and 55% to 34% when using the local currencies. Specifically, the time periods in which FTSE 100 is strongly long-run Granger caused by S&P 500, namely periods 1–4, 13, 23, 33–34, all accompany economic recession or financial shocks in the US market, whether we measure them in common or local currency terms. In contrast, the significant negative error correction coefficients are found as an evidence of long-run Granger causality running from FTSE 100 to S&P 500 during periods 1, 4, 15–16 40, i.e., early 1980s recession in the UK, UK market’s “Black Wednesday” currency crisis in 1992, and the subsequent 1992–93 European currency crisis, significantly Granger caused the US stock market in the long run. Furthermore, significantly directional long-run Granger causality between S&P 500 and FTSE 100 are found during the early 1980s recession in the US and UK, following the 1993 economic recovery of US and UK, the early 1990s recession in the US and UK (only using GBP/GBP and local currencies), the early 2000s recession in the US (only using GBP/GBP and local currencies). Meanwhile, the statistical results in Table 7 show that the dynamic error correction coefficients vary over time. In most of the time periods, the coefficients that show evidence of long-run Granger causality running from S&P 500 to FTSE 100 are stronger than the reverse direction, when measured in USD/USD (average values of 0.387 vs. 0.366) and local currencies (average values of 0.429 vs. 0.377), which indicates that the US stock market is more influential than the UK market. However, contrasting results are found when we use GBP/GBP (average values of 0.336 vs. 0.349). Moreover, the strongest coefficients for the S&P 500 long-run Granger causes FTSE 100 is 0.970 (using USD/USD during period 42), 0.895 (using GBP/GBP during period 42), and 0.926 (using USD/GBP during period 34). It should be noted that since the high volatility during the August 2011 stock market fall and the 2007–09 global financial crisis, the shock of US stock market exerts a significant leadership toward UK market.

The statistical significant and negative adjustment coefficients for S&P 500 and EURO STOXX 50 in Fig 13(a)–13(c) provide evidence of long-run causal relationship running for S&P 500 to EURO STOXX 50 (shown with a blue bar), from EURO STOXX 50 to S&P 500 (shown with a yellow bar) from 1998 to 2015 calculated using the same and local currencies, respectively. From Table 5, we find that the proportion of period in which the S&P 500 long-run Granger causes EURO STOXX 50 is stronger than the reverse, namely 56% to 53% when using USD/USD and 50% to 50% when using EUR/EUR, and 56% to 50% measured in the local currencies. Furthermore, the time periods in which the S&P 500 strongly long-run Granger causes EURO STOXX 50 are particularly during the 1999 Kosovo war, the 2002 stock market downturn, the collapse of the US housing bubble, the 2007–09 global financial crisis, the 2010 European debt crisis, the 2015–16 US stock market sell-off, all of which are accompanied by economic, financial or political shocks in the US market. However, the reverse direction that EURO STOXX 50 long-run Granger causes S&P 500 is observed during the burst of the 2000 dot-com bubble, the beginning of US housing bubble period, from the early 2000s recession in the US to the 9/11 attack and war in Afghanistan, the beginning period of the US housing price bubble, the 2010 European debt crisis, the period that second round of QE implementation in the UK. It should be noted that, when measured in EUR/EUR, there is strongly long-run Granger causality running from the EURO STOXX 50 to S&P 500 after the Lehman Brother collapse in Sept. 2008 since the significant depreciation of Euro against US dollars, resulting in money inflows and investment shock in the Eurozone stock markets and causes changes in S&P 500. Moreover, Table 7 displays the average error correction coefficients between the S&P 500 and EURO STOXX 50, using both the same and local currency terms, and the findings further prove that the long-run Granger causality between S&P 500 EURO STOXX 50 is similar, with average values of 0.441 vs. 0.416 in USD/USD, 0.339 vs. 0.368 in EUR/EUR and 0.504 vs. 0.511 in USD/EUR, respectively. The maximum error correction coefficients for the S&P 500’s causes EURO STOXX 50, 1.668 using USD/USD in period 39, 1.407 using EUR/EUR in period 29, and 1.332 using local currencies in period 29, are associated with the 2009 Dubai debt standstill and the 2002 stock market downturn.

Finally, Fig 14(a)–14(c) show the estimation of dynamic adjustment coefficients for the ECM-based long-run Granger causality for FTSE 100 and EURO STOXX 50, from 1998 to 2015 in both common and local currency terms, respectively. The statistical significant and negative adjustment coefficients provide an evidence of long-run Granger causal relationship running for FTSE 100 to EURO STOXX 50 (shown with a blue bar), from EURO STOXX 50 to FTSE 100 (shown with a yellow bar) respectively. As shown in Table 6, the proportion of period in which the FTSE 100 long-run Granger causes EURO STOXX 50 is much more compared with the causality running from EURO STOXX 50 to FTSE 100, namely 44% to 31% when using GBP/GBP, 34% to 25% when using EUR/EUR and 47% to 34% with local currency terms. Moreover, the time periods in which the FTSE 100 strongly long-run Granger causes EURO STOXX 50 are especially during the 1999 Kosovo war, the 2002 stock market downturn, the collapse of US housing bubble, the 2007–09 global financial crisis, the 2010 European debt crisis, the US recession of Dec 2007–Jun 2009, the US QE2 from November 3th, the 2010 to June 30th, 2011, the 2015–16 US stock market selloff. However, the reverse Granger causal direction that EURO STOXX 50 long-run Granger causes FTSE 100 is during the 9/11 Attacks, the 2001 US war in Afghanistan, the August 2011 US stock market fall, during the 2013 US debt-ceiling crisis, the implementation of US QE3 and tapering announced and UK QE2, respectively. Next, from the average error correction coefficients between the FTSE 100 and EURO STOXX 50 shown in Table 7, we notice that the EURO STOXX 50 long-run Granger causes FTSE 100 is slightly stronger than the reverse direction, with average values of 0.498 vs. 0.425 (GBP/GBP), 0.479 vs. 0.504 (EUR/EUR), and 0.553 vs. 0.524 (local currency terms). The strongest coefficients by which the long-run Granger causality running from FTSE 100 to EURO STOXX 50 is 1.225 (with GBP/GBP in period 29), 1.154 (with EUR/EUR in period 29) and 0.930 (with local currency terms in period 29). What is more, the strongest coefficients of the EURO STOXX 50 long-run Granger causality FTSE 100 are 1.119 (with GBP/GBP in periods 27–28), 1.233 (with EUR/EUR in periods 27–28), and 1.050 (with local currency terms in period 42). The results reveal that, since various bilateral trade and economic cooperation agreements exist between the US, UK and the Eurozone markets, the 9/11 attack, the 2001 US war in Afghanistan, and the August 2011 stock market fall resulting in significantly long-run Granger causal relation between FTSE 100 and EURO STOXX 50.

### Summary results of dynamic correlation, cointegration and ECM-based long-run Granger causality analysis

From the results of dynamic correlation, cointegration and ECM-based long-run Granger causality analysis between the S&P 500 and FTSE 100, S&P 500 and EURO STOXX 50, and FTSE 100 and EURO STOXX 50 over 1980–2015 in both common and local currencies terms, the following similarities are derived. As shown in Figs 2 and 6–11, the dynamic correlation and cointegration analysis between all pairs of stock market indices become stronger and more deeply integrated with each other when they are associated with economic, financial and political shocks. However, the decreasing, weaker correlation and cointegration evolving over time have been found during the bull market or the recovery of the stock market after serious shocks. Specifically, identifying the similarities between dynamic correlation and ECM-based long-run Granger causality provides more interesting results not only for the interaction detection but also for the directed causal relations.

The dynamic correlation analysis highlights the interactions between US and UK stock markets tend to increase significantly during: 1) the early 1980s recession of the US, the 1984–85 UK miners’ strike, the 1990 Gulf War, both associated with bidirectional long-run Granger causality running between US and UK stock markets; 2) the 1987 “Black Monday” stock market crash, the 2002 stock market downturn, the 2007 sub-prime mortgage crisis, the 2011 US debt-ceiling crisis, associated with long-run Granger causality running from the S&P 500 to FTSE 100; 3) the 1992–93 European currency crisis, before the 1997 Asian financial crisis, with long-run Granger causality running from FTSE 100 to S&P 500. In contrast, the significantly decreasing correlation between S&P 500 and FTSE 100 are observed during: 1) the 1982 economic recovery of the US and the UK, the 1994 Mexico peso crisis, accompanied with long-run Granger causality running from the US to the UK stock market; 2) the 1992–93 European currency crisis, the period of the US Dot-com bubble, the period of 2004–2006 US housing price bubble, with long-run Granger causality running from the UK to the US stock market.

In terms of the correlation dynamics across the US and Eurozone stock markets tend to increase significantly during: 1) the bear market between post 2001 and 2003, the US recession from December 2007 to post 2008, the Lehman brother collapse in September 2008, the 2015-16 US stock market sell-off, associated with long-run Granger causality running from the S&P 500 to the EURO STOXX 50; while during: 2) the 2000 dot-com bubble burst, the beginning of US housing bubble from 2004—05, the August 2011 stock market fall, all associated with long-run Granger causality running from the EURO STOXX to the S&P 500. In contrast, we can observe the gradually decreasing correlation during the periods after the introduction of Euro and the 1999 Kosovo war, the beginning of 2007, both associated with significant magnitude of long-run Granger causality from the S&P 500 to the EURO STOXX 50; while long-run Granger causality from the EURO STOXX 50 to the S&P 500 during the second round of US QE policy implementation.

By observing the dynamic correlation and ECM-based long-run Granger causality of the FTSE 100 and EURO STOXX 50, all increasing correlation accompanied with significantly stronger long-run Granger causality in both direction during: 1) the bear market between post 2001 and 2003 with FTSE 100 long-run Granger causes EURO STOXX; 2) the 9/11 Attack, the 2001 US war in Afghanistan and the August 2011 stock market fall with significantly long-run Granger causality running from the EURO STOXX 50 to the FTSE 100. On the contrary, the decreasing correlation associated with direction causal relations during the introduction of the Euro, the 1999 Kosovo war and the 2005—06 US housing price bubble, the US QE2, the EU QE during 2015–16, both associated with long-run Granger causality running from the FTSE 100 to the EURO STOXX 50, respectively. However, during the implementation of QE in the US (QE3 and tapering policies announced), the EURO STOXX 50 significantly long-run Granger causes the FTSE 100 with decreasing correlation.

We summary the similarities and differences from dynamic correlation, cointegration and ECM-based long-run Granger causality analysis of each pair of developed stock markets of the US, UK and Eurozone as follows:

- During the periods of economic, financial and political episodes, the degree of dynamic correlation, cointegration and ECM-based long-run Granger causality between the pairs of stock market indices increased significantly in all cases. However, during the bull market and recovery period of the stock market after shocks, the correlation decreased gradually associated with weaker integration and long-run Granger causality. In particular, there is stronger and more significant interactions, Granger causal relations between the stock market indices when they are both measured in local currency terms.
- The dynamic correlation analysis ascertains the degree of co-movement between stock markets based on synchronous changes, which might miss long-run relationships occurring on a long time scale. Since the cointegration tests capture the long-run equilibrium relations between two stock market indices that are cannot deviate too far away from each other in the long term, the dynamic cointegration between pairs of stock markets is more persistent than the dynamic correlation associated with economic, financial and political episodes. Furthermore, the ECM tests to examine whether returns of one market influence another based on the existed long-run cointegration, which could reflect the direction of the long-run Granger causality between stock market indices efficiently.

Finally, the understanding on the dynamic *integration* and *causality* between the various national stock markets is important since it has direct impact on investors’ investment strategy particularly those that involves cross-border investments. A combination of not perfectly correlated stock markets gives the investor an opportunity to gain from portfolio diversification. For investors with longer time horizons, the benefit of international diversification can be attained if one country’s stock market is not cointegrated with other country’s stock market [44]. However, our empirical findings indicate that the presence of the increasing correlation, cointegration and long-run Granger causality between the local stock markets with foreign stock markets during the economic, financial and political shocks, may limit potential portfolio diversification benefits in the sample stock markets.

## Conclusion

In this paper, by combining the rolling-window technique with correlation, cointegration and ECM tests, we explore the dynamic *integration* and *causality* between each pair of US, UK, and Eurozone stock markets from January 1980 to December 2015 under the impact of a series of economic, financial and political shocks. Specifically, we measure those time-varying symmetric and asymmetric interactions under the same currencies and under local currencies to comprehensively analyze how the exchange rates fluctuation affects the integration and linkages between stock market indices over time. Besides, the similarity and difference between the integration and causality are studied.

The findings obtained indicate that the degree of short-term correlation, long-term cointegration and ECM-based long-term Granger causality between all pairs of stock market indices both changed over time. Especially, stronger interactions and causality when measured in local currency terms than used in common currencies. The dynamic correlation analysis ascertains the degree of co-movement between the US, UK and Eurozone stock markets based on stationary returns, and highlights the interactions between stock markets tend to increase during economic, financial and political shocks over 1980–2015. However, decreasing correlations were found during the bull market and the recovery of the stock market after shocks. Similarly, the existence of long-run cointegration between each pairwise of stock markets is more significant during times of economic, financial and political episodes, whereas the weaker cointegration varied over time has been found during the bull market or the recovery of the stock market after those “extreme events”. In particular, the strongest and most persistent cointegration exists between US, UK and Eurozone stock markets are during the 2007–09 global financial crisis.

Furthermore, the ECM-based long-run Granger causality which exacts from the existed cointegration relationships reveals the directed dynamic causal relation between pairwise stock markets of US, UK and Eurozone from 1980 to 2015. Specifically, we found that associated with increasing correlation evolved with time, the US stock market long-run Granger caused the UK and Eurozone markets during the economic, financial and political episodes happened in the US market, for example, during the 1987 “Black Monday” stock market crash, the 2002 stock market downturn, the 2007 sub-prime mortgage crisis and the Lehman Brother collapse in September 2008, etc. In contrast, the UK and Eurozone markets cause the US market especially during the 1992–93 European currency crisis, the 2000 dot-com bubble burst and the beginning of the US housing bubble from 2004–05, etc. In particular, there is significantly stronger long-run Granger causality from the UK to Eurozone markets during the bear market between post 2001 and 2003, meanwhile, Eurozone stock markets lead UK market during the periods of the 9/11 Attack, the 2001 US war in Afghanistan and the August 2011 stock market fall all accompanied with increasing correlation, respectively. On the other hand, with the decreasing correlation over time, the US market has remained dominant in leading the information transmission to UK and Eurozone markets during the 1982 economic recovery of US and UK, the 1994 Mexico peso crisis, the periods after introduced the Euro, the 1999 Kosovo war and the beginning of 2007. However, we find the unidirectional causality from the UK, Eurozone markets to the US market during the 1992–93 European currency crisis, the period of the US Dot-com bubble, the period of 2004–06 US housing price bubble and the US QE2 policy implementation. The obtained results further show that during the introduction period of Euro, the 1999 Kosovo war, the 2005–06 US housing price bubble, the US QE2, the EU QE during 2015–16, there is long-run Granger causality from UK to Eurozone markets, while the reverse causality could be observed during the implementation of QEs in the US (QE3 and tapering announced).

To conclude, our results suggest that the potential for diversifying risk by investing in the US, UK and Eurozone stock markets is limited during the periods of economic, financial and political shocks. Testing for cointegration and any changes in it over time is crucial since, if cointegration does not hold, it indicates that the markets are not linked and no Granger causality in the long run and therefore it is possible to gain from diversification. As for the dynamic correlation, the lower correlation between pairs of stock markets will be beneficial to investors.
